# Maintaining their genetic distance: Little evidence for introgression between widely hybridizing species of *Geum* with contrasting mating systems

**DOI:** 10.1111/mec.14426

**Published:** 2017-12-09

**Authors:** Crispin Y. Jordan, Konrad Lohse, Frances Turner, Marian Thomson, Karim Gharbi, Richard A. Ennos

**Affiliations:** ^1^ Ashworth Laboratories Institute of Evolutionary Biology University of Edinburgh Edinburgh UK; ^2^ Ashworth Laboratories Edinburgh Genomics Edinburgh UK

**Keywords:** coalescent, *Geum*, hybridization, introgression, natural selection

## Abstract

Within the plant kingdom, many genera contain sister lineages with contrasting outcrossing and inbreeding mating systems that are known to hybridize. The evolutionary fate of these sister lineages is likely to be influenced by the extent to which they exchange genes. We measured gene flow between outcrossing *Geum rivale* and selfing *Geum urbanum*, sister species that hybridize in contemporary populations. We generated and used a draft genome of *G. urbanum* to develop dd‐RAD data scorable in both species. Coalescent analysis of RAD data from allopatric populations indicated that the species diverged 2–3 Mya, and that historical gene flow between them was extremely low (1 migrant every 25 generations). Comparison of genetic divergence between species in sympatry and allopatry, together with an analysis of allele frequencies in potential parental and hybrid populations, provided no evidence of contemporary introgression in sympatric populations. Cluster‐ and species‐specific marker analyses revealed that, apart from four early‐generation hybrids, individuals in sympatric populations fell into two genetically distinct groups that corresponded exactly to their morphological species classification with maximum individual admixture estimates of only 1–3%. However, we did observe joint segregation of four putatively introgressed SNPs across two scaffolds in the *G. urbanum* population that was associated with significant morphological variation, interpreted as tentative evidence for rare, recent interspecific gene flow. Overall, our results indicate that despite the presence of hybrids in contemporary populations, genetic exchange between *G. rivale* and *G. urbanum* has been extremely limited throughout their evolutionary history.

## Abstract 2

https://doi.org/10.1111/mec.14444

## 1 INTRODUCTION

A key factor influencing the evolutionary trajectory of lineages is their level of genetic exchange with related taxa (Abbott, Barton, & Good, 2016; Coyne & Orr, 2004); for example, genetic exchange can introduce adaptive mutations (e.g., Paoletti, Buck, & Brasier, 2006; Whitney, Randell, & Rieseberg, 2010) or restrict the lineages’ independent evolution. Self‐fertilization (“selfing”) frequently evolves from outcrossing plant species, yielding many sister species pairs with contrasting mating systems (Barrett, Arunkumar, & Wright, 2014; Igic, Bohs, & Kohn, 2006; Stebbins, 1957; Wright, Kalisz, & Slotte, 2013). Contemporary populations display widespread evidence for hybridization between outcrossing and inbreeding sister species (e.g., *Mimulus* (Brandvain, Kenney, Flagel, Coop, & Sweigart, 2014; Kenny & Sweigart, 2016; Vickery, 1978), *Rhinanthus* (Ducarme, Vrancken, & Wesselingh, 2010), *Centaurium* (Brys, Vanden Broeck, Mergeay, & Jacquemyn, 2014), but we have little understanding of whether this leads to significant introgression between them.

Unlike outcrossing species pairs, members of pairs with contrasting mating systems differ in the potential for introgression and the processes that cause it. Recurrent backcrossing of hybrids to the outcrossing parent can allow genetic exchange from inbreeding to outcrossing lineages. However, introgression from outcrosser to inbreeder is likely to be rare by backcrossing to inbreeding parents, at least for animal‐pollinated species, although may increase if hybrids self‐fertilize. That said, introgression may be of most importance to inbreeders, whose evolutionary potential is otherwise compromised by low genetic diversity and effective recombination rates (Arunkumar, Ness, Wright, & Barrett, 2015; Charlesworth, Morgan, & Charlesworth, 1993, 2003; Glémin & Ronfort, 2013).

A number of factors potentially limit introgression. Evolution of a selfing syndrome in inbreeders, conspecific pollen precedence and ecological differentiation between the species creates prezygotic barriers to introgression (Fishman & Wyatt, 1999; Sicard & Lenhard, 2011), while divergence of genomes under different mating systems, chromosomal and ploidy differences, and genetic adaptation of the species to distinct habitats will reduce the fitness of recombinant hybrids and generate postzygotic reproductive barriers (Hu, 2015; Wright, Ness, Foxe, & Barrett, 2008). Thus, the potential for introgression between outcrossing and inbreeding sister lineages may not be realised. The importance of introgression can only be determined through empirical estimates of introgression rates. Here, we use RAD data to quantify historical and contemporary introgression between two hybridizing sister species with contrasting mating systems in the plant genus, *Geum* (Smedmark, Eriksson, Evans, & Campbell, 2003).

**Figure 1:**
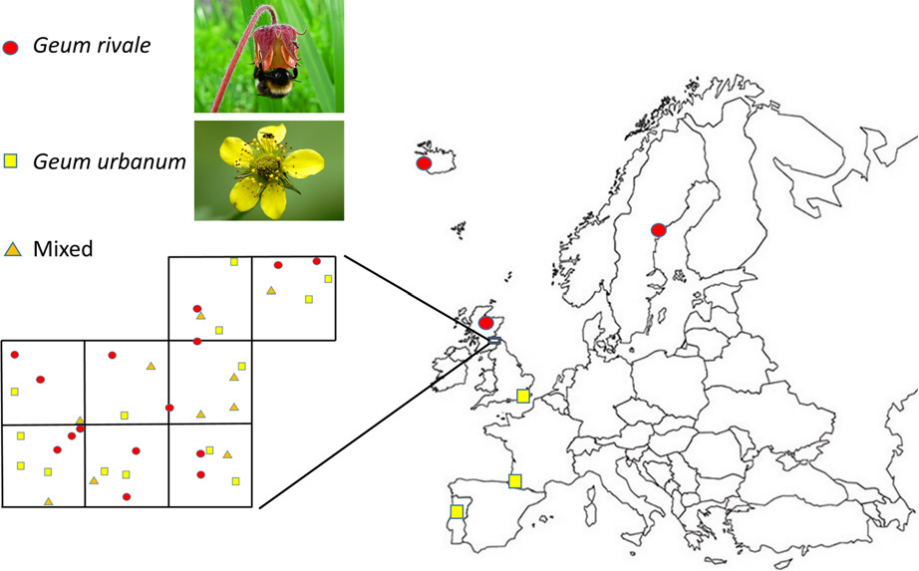
Locations of allopatric populations (large map) of *Geum rivale* and *Geum urbanum* and detailed distribution of 39 sympatric populations in eight 10 km × 10 km squares (inset) [Colour figure can be viewed at http://wileyonlinelibrary.com]

The study species (Figure 1) comprise *Geum rivale* (outcrossing rate *t* = 0.8), which possesses a pendulous flower adapted to bee pollination and typically occupies open, moist habitats, and inbreeding *Geum urbanum* (*t* = 0.15), which bears erect flowers adapted to fly pollination and grows in shaded, well‐drained sites (Ruhsam, Hollingsworth, Squirrell, & Ennos, 2010; Taylor, 1997a, 1997b). Both species are perennial and ancient allohexaploids (2*n* = 42; Smedmark et al., 2003). Populations of *G. rivale* that lack *G. urbanum,* and have likely done so for a long period (“allopatric” populations), occur in tall herb montane communities in the UK, and at high latitude sites elsewhere in Europe. Allopatric *G. urbanum* populations occur in the south‐east of the UK, where rainfall is low (<650 mm/year), and in the south of its continental European range (Taylor, 1997a, 1997b). Elsewhere in the UK and continental Europe, the species occur sympatrically, either alone or in mixed populations.

The highly fertile F1 hybrid *G. × intermedium* occurs in mixed populations (typically wet woodlands; Waldren, Etherington, & Davies, 1989), often within hybrid swarms (Taylor, 1997a) that occur commonly throughout the UK. F1 hybrids have flowering times that overlap those of both parent species (Ruhsam, Hollingsworth, & Ennos, 2011). Previous analyses of a hybrid swarm revealed the presence of both F1 hybrids and early‐generation backcrosses to *G. rivale*, which produced fertile seeds by both outcrossing and selfing (Ruhsam, Hollingsworth, & Ennos, 2013; Ruhsam et al., 2011). Later generation recombinant offspring expressed no detectable fitness reduction in a benign environment, but were absent from the hybrid swarm population (Ruhsam et al., 2013). Thus, the mating events necessary for introgression between *G. rivale* and *G. urbanum* occur, but gene exchange has not been quantified.

We adopt a comparative genomic approach to quantify both historical and contemporary gene exchange between the two *Geum* taxa. We first generate a draft genome of the inbreeding species, *G. urbanum*, which we use to develop SNP markers common to the two species. Then, we score these SNPs in allopatric populations likely to have been geographically isolated for the past 5 kyr (McVean & Ratcliffe, 1962). We apply coalescence analysis to these data to determine the age of the taxa and estimate historical introgression rates to contextualize our analysis of contemporary introgression. We also use allopatric samples to identify species‐specific diagnostic SNPs.

To test for recent gene flow between the *Geum* taxa, we compare allopatric populations with samples from a broad area of sympatry (see Section 2). Here, our analyses focus on detecting long‐term introgression, because formation of early hybrids is known. Based on hybrid classes found within a hybrid swarm (Ruhsam et al., 2013), we predict that, if introgression occurs in the area of sympatry, gene flow will primarily occur from the inbreeder (*G. urbanum*) to the outbreeder (*G. rivale*). We (i) test whether allele frequencies of a potential hybrid population disproportionately reflect allele frequencies of one likely parental population (suggesting introgression; f3 test: Patterson et al., 2012); (ii) test whether genetic differentiation between species in allopatry exceeds that in sympatry, as expected with introgression; (iii) apply a clustering analysis of multilocus SNP genotypes to test whether admixture of *G. rivale* individuals exceeds that of *G. urbanum*; (iv) score sympatric samples using species‐diagnostic SNPs to quantify introgression for individuals. Finally (v), we relate estimates of an individuals’ hybridity to its morphology to test whether a plant's morphology reflects its genotypic hybrid status and to test whether morphology varies with focal RAD loci.

## 2 MATERIALS AND METHODS

### 2.1 Sampling

#### 2.1.1 Sampling of allopatric populations

Four *G. rivale* individuals were sampled from each of three high elevation tall herb communities located on three distinct Scottish mountain ranges above the altitudinal limits of *G. urbanum* (Table S1; Taylor, 1997b). One individual was also sampled from a single population in each of northern Sweden and Iceland where *G. urbanum* is absent (Table S1; http://linnaeus.nrm.se/flora).

Two *G. urbanum* plants were sampled from each of 10 populations in south‐east England (Table S1), a region where *G. rivale* is absent due to lack of suitable habitats (Preston, Pearman, & Dines, 2002). Single individuals of *G. urbanum* were also sampled from two areas in Europe where *G. rivale* does not occur, namely Portugal and south‐west France (Table S1; http://linnaeus.nrm.se/flora). 0.5 g of young leaf tissue was removed from sampled plants and stored dry in silica gel prior to DNA extraction.

#### 2.1.2 Sampling of sympatric populations

We aimed to test whether recent introgression affects *G. rivale* and *G. urbanum* beyond hybrid swarms. Hence, we sampled sites (a) with both species present and (b) where each species lived individually. However, unlike “allopatric” populations (see above), the sites with only one *Geum* species had habitat characteristics that could allow introgression by interpopulation gene flow or could have allowed both species to co‐occur in the recent past. Hence, we broadly define “sympatric” populations as localities where (in the absence of selection) recent introgression should be possible. The Botanical Society of Britain and Ireland (BSBI) database was searched for the location of sites recorded as containing *G. rivale* alone (15), *G. urbanum* alone (14) or both taxa (10) in eight contiguous 10 km × 10 km squares within vice county 81 in the British Isles (Berwickshire) (Figure 1, Table S2; http://bsbidb.org.uk/maps/). Plant collections were made by taking cuttings of plants (a minimum of 5 m apart) from these sites in August 2013. At single taxon sites, two individuals were randomly sampled. At mixed sites, cuttings were collected from four randomly selected *Geum* plants located across the full range of ecological conditions present at the site. Initial field identification was to genus only. Ninety‐six of the 98 cuttings were rooted successfully in 20‐cm‐diameter pots containing commercial potting compost. Newly emerging leaves (0.1 g/plant) were frozen (−80°C) prior to DNA extraction. Plants were overwintered outside in a randomized array in Edinburgh to induce flowering.

### 2.2 Development of SNP markers via ddRAD

#### 2.2.1 Overall strategy

The genomes of these ancient hexaploid (Smedmark et al., 2003) species are large (1.6 and 1.2 Gb in *G. urbanum* and *G. rivale,* respectively). Therefore, to ensure adequate depth of coverage, we adopted the ddRAD technique that allows modulation of the number of RAD tags generated per genome (Peterson, Weber, Kay, Fisher, & Hoekstra, 2012). To maximize the chance of distinguishing homologs and paralogs, we used paired‐end (PE) sequencing yielding long reads and generated a draft genome of the largely homozygous inbreeding species against which to map our ddRADtag sequences.

#### 2.2.2 DNA extraction

DNA was extracted from dried samples using a modification of the CTAB protocol of Doyle and Doyle (1990), while fresh and frozen material was extracted using the DNeasy plant mini prep kit (Qiagen) following the manufacturer's protocol.

#### 2.2.3 Development of draft genome

To develop the draft genome, we identified the most homozygous individual (10 microsatellite loci; Arens, Durka, Wernke‐Lenting, & Smulders, 2004) among 10 additional samples from the Punnetts Town population (Table S1). From this, we extracted 17 μg of DNA from 150 mg of young leaves. Details of the genome sequencing and assembly, which were conducted by Edinburgh Genomics, are given in the Supporting Information.

#### 2.2.4 ddRAD library prep and sequencing

DNA quality was evaluated using the E‐Gel Precast Agarose Electrophoresis System (ThermoFisher Scientific), and samples quantified using the Qubit dsDNA BR Assay Kit (ThermoFisher Scientific). Sample preparation, library construction and PCR amplification for double‐digest RAD sequencing were modified from Peterson et al. (2012; for full details see Supporting Information). Superpools of PCR products were sequenced either on an Illumina hiseq 2500 using 125 base pair PE reads in high output mode (v4 chemistry) or an Illumina miseq using 150 base PE reads (v2 chemistry).

#### 2.2.5 Quality filtering and preparation of reads from ddRAD

The bioinformatics and analysis pipeline for all ddRAD data are summarized in Figure S1. To match the read lengths produced by miseq and hiseq Illumina technologies for ddRAD analyses, we used fastx trimmer (http://hannonlab.cshl.edu/fastx_toolkit/) to trim miseq reads to 125 bp (stacks requires reads of equal length). We de‐multiplexed and filtered reads for quality using process_radtags (stacks v 1.21; Catchen, Amores, Hohenlohe, Cresko, & Postlethwait, 2011; Catchen, Hohenlohe, Bassham, Amores, & Cresko, 2013). This removed reads with an uncalled base, and/or an average quality score <20 over a sliding window comprising 15% of a read. Finally, we trimmed reverse reads to 117 bp with fastX trimmer to produce forward and reverse reads of equivalent length after the 8‐bp barcode was removed from the forward reads.

#### 2.2.6 Aligning reads to draft genome

Mapping ddRAD sequences from the two *Geum* species must account for divergence between species to allow reads from both species to map to the *G. urbanum* draft genome. However, allowing an excessive number of mismatches can cause reads to map to multiple locations in ancient polyploid taxa, like *Geum*. We explored the effect of varying the number of allowed mismatches (*M*) on read mapping for one allopatric individual from each species (Punnetts Town, *G. urbanum*; Ben Lui, *G. rivale*; both sequenced with Illumina hiseq).

For a range of *M* values, we used gsnap (version 2014‐12‐16; Wu & Watanabe, 2005) to map reads to the draft genome. We required that reads map uniquely, specified an indel penalty of two, and did not allow terminal alignments. For the *G. urbanum* sample, between 64.86% and 67.74% of PE ddRAD reads mapped as proper pairs when we allowed one to nine mismatches (*M* = 1–9); however, the percentage of read pairs that mapped jumped to 82.84% for *M* = 10. Similarly, the percentage of *G. rivale* PE reads that mapped in proper pairs increased gradually from 39.21% to 58.16% over *M* = 2 to *M* = 9, with a large increase to 79.93% for *M* = 10. Therefore, the structure of the *Geum* genome (e.g., due to polyploidy and/or repetitive sequences) appears to present different environments for mapping ddRAD data at a threshold genetic diversity of *c*. 8.55% (i.e., 10/117 × 100, where 117 equals the read length). To avoid mapping to paralogous regions while maximizing the number of *G. rivale* reads correctly mapped to the heterospecific draft genome, we specified *M* = 6 throughout.

#### 2.2.7 Assembling radtags and calling SNPs

We used stacks’ ref_map pipeline (v 1.21; Catchen et al., 2011, 2013; Hohenlohe et al., 2010) to assemble the aligned reads into radtags and the rxstacks module to correct genotype calls based on population‐level genotype information using a bounded (error rate) model (setting conf_lim = 0.25 and bound_high equal = 0.1, see http://catchenlab.life.illinois.edu/stacks/manual/).

#### 2.2.8 Identifying and filtering for paralogs

Ancient hexaploidy complicates analyses of *Geum* because reads from paralogous genome regions could map to identical sites. To identify genome regions that potentially attract paralogous reads, we used stacks’ populations module to calculate observed heterozygosity and *F*_IS_ at each identified SNP. Thereafter, custom scripts were used to collate data and restrict further analysis to scaffolds free of SNPs that exhibited either excess heterozygosity (>0.5) or negative *F*_IS_. The heterozygosity and *F*_IS_ calculations *for this purpose only* considered UK allopatric *Geum* samples that comprised four individuals from each of three Scottish “allopatric” *G. rivale* populations (12 *G*. *rivale* individuals), and one randomly chosen individual from each of the 10 Southern England *G. urbanum* populations (Table S1). Nucleotide‐level heterozygosity and *F*_IS_ were calculated separately for *G. rivale* and *G. urbanum*; we assumed each set of samples was from a single, large UK population, despite population structure being evident in both species (see Section 2.1). Population structure reduces the number of heterozygotes compared to a single panmictic population: therefore, our filters should preferentially remove genomic regions with excess heterozygosity due to paralogy.

### 2.3 Population genetic analysis of allopatric populations

#### 2.3.1 Patterns of polymorphism within and between species

We characterized polymorphism within and between the *Geum* species using the 12 allopatric UK *G. rivale* samples plus one each from Iceland and Sweden, and 10 UK *G. urbanum* samples (one from each allopatric UK population) plus one each from France and Portugal (Table S1). We used stacks’ populations module to identify SNPs that are polymorphic within at least one species or show a fixed difference between species.

#### 2.3.2 Inbreeding coefficients and population differentiation

We applied the stacks’ populations module to estimate the inbreeding coefficients within each population (*F*_IS_) and measure differentiation among populations (*F*_ST_) for each species using data from the three allopatric *G. rivale* populations in Scotland (four individuals/pop) and the 10 allopatric *G. urbanum* populations in England (two individuals/pop). Estimates of *F*_IS_ and *F*_ST_ only considered radtags present in both species and in all individuals of a given analysis.

#### 2.3.3 Identification of species‐specific SNPs

We used alternately fixed SNPs in allopatric populations of the two taxa (identified in Section 3 above) to provide an initial list of species‐specific SNPs. To minimize linkage between diagnostic SNPs and obtain an estimate of introgression across the whole genome, we selected one (the first) alternately fixed SNP per scaffold for the introgression analysis. However, we note that using the first SNP per scaffold biases our data against (larger) well‐assembled scaffolds. Therefore, we repeated our analyses of introgression using all available species‐diagnostics SNPs and obtained qualitatively identical results (not shown).

#### 2.3.4 Coalescent analysis of gene flow during lineage divergence

We used an analytic likelihood framework to assess the support for alternative models of divergence between *G. rivale* and *G. urbanum* with and without gene flow. The method is described in Lohse, Chmelik, Martin, and Barton (2016) and is based on the joint frequency of mutations in blocks that are assumed to be unlinked and neutrally evolving (we consider the likely effects of violating these assumptions in the Section 2.1.1). Briefly, the analysis is based on a single diploid individual for each species and considers the blockwise site‐frequency spectrum, that is, the joint frequencies of four polymorphism types (as in “Section 3”, above) in short blocks of sequence: (i) heterozygous sites exclusive to *G. rivale*, (ii) heterozygous sites exclusive to *G. urbanum*, (iii) heterozygous sites shared by both species and (iv) fixed differences between species. We counted these site types within 117 bp radtags (block), and treated each radtag as an independent block. For randomly mating populations, the polymorphisms at each block represent an independent outcome of the coalescent process, which is a function of the species’ history. As stacks ignores RAD tags that are monomorphic, we conditioned the likelihood on only observing variable blocks by normalizing the probabilities of blockwise mutational configurations by 1 − p_IBS, where p_IBS is the probability of identity in state for a block.

We primarily wish to test for historical introgression between *G. rivale* and *G. urbanum*. Therefore, we compared three models: (1; “div_2_”) species diverged at time *T* with no introgression, (2; “IM_u→r_”) a history of divergence with gene flow from *G. urbanum* to *G. rivale* and (3; “IM_r→u_”) divergence with gene flow from *G. rivale* to *G. urbanum* (four parameters). All models assume instantaneous species divergence and a constant *N*_e_ within taxa. We also constrained the effective population size of the ancestor (*N*_anc_) to equal that of *G. rivale* (the ancestor of *G. rivale* and *G. urbanum* is most likely outcrossing given the rarity of transitions from self‐compatibility to self‐incompatibility; Igic et al., 2006). To capture the decrease in effective population size (*N*_e_) expected to result from the transition to inbreeding (Charlesworth & Wright, 2001), we allowed a different *N*_e_ for *G. urbanum*. Migration was modelled as a constant rate *M* = 4*N*_anc_ × *m* individuals per generation where *m* = probability of migration each generation. We converted estimates of *T* into years using *t* = *T* × 2*N* × *g* where *g* is generation time and *N* = θ/4μ. We assumed a mutation rate estimated for *Arabidopsis thaliana*, μ = 7 × 10^−9^ (Ossowski et al., 2010), and an average generation time across the two species of 3 years (Taylor, 1997a, 1997b; generations are likely longer in *G. rivale* than in *G. urbanum*).

Inbreeding complicates this analysis because it reduces genetic diversity within individuals relative to the population level. To minimize the confounding effect of inbreeding on the analyses, we initially chose the most outbred individuals for analysis. We used a recently outcrossed *G. urbanum* individual (see Section 2.1; population Mill Wood), which had genetic diversity (π = 0.0009 ± 0.0001) similar to the UK allopatric *G. urbanum* populations as a whole (see Section 2.1), and so may adequately represent the diversity of this species in the UK. “Leaky” self‐incompatibility also introduces variation in inbreeding among individuals of *G. rivale* (Ruhsam et al., 2010). Therefore, we conducted separate analyses that paired the outbred *G. urbanum* Mill Wood individual with three allopatric UK samples of *G. rivale* that span a range of heterozygosity to examine how inbreeding in *G. rivale* affects our conclusions.

Maximum‐likelihood estimates under each model were obtained in mathematica v. 10.2 (see Data S2). To estimate 95% CI for *M* and *T*, we obtained discretized marginal support (logarithm of the likelihood) curves for these parameters (maximizing the likelihood for all other parameters).

### 2.4 Population genetic comparison of allopatric and sympatric populations

#### 2.4.1 f3 tests for introgression

We calculated the f3 statistic (Patterson et al., 2012) to test for introgression into either the sympatric *G. rivale* or *G. urbanum* populations; f3 compares allele frequencies in two potential source populations (a, b) and a potential hybrid population (c). A significantly negative value of f3 indicates introgression (Patterson et al., 2012).

We calculated f3 as the mean of (c−a) (c−b) over all SNPs (Patterson et al., 2012). Allele frequencies (a) and (b) included only UK allopatric *G. rivale* and *G. urbanum* samples (12 and 10 samples, respectively), because we expected these populations (i.e., not continental European populations) to most contribute to introgression in Berwickshire. As we are interested in detecting long‐term introgression, we omitted four obvious early‐generation hybrids in Berwickshire, described in the Section 2.1. We considered both Berwickshire *G. rivale* and *G. urbanum* populations as potentially admixed (i.e., populations “c”). All allele frequency estimates involved at least 10 individuals per population.

We bootstrapped our data at the level of scaffolds to generate 95% confidence intervals for the f3 estimates. We resampled scaffolds with replacement and calculated f3 for 2,000 bootstrapped data sets and used the 2.5% and 97.5% quantiles as estimates of 95% CI's of f3.

#### 2.4.2 Genetic differentiation between taxa in allopatry and sympatry

To test whether potential gene exchange resulted in reduced differentiation between the species in sympatry, we calculated genetic differentiation *d*_*xy*_ = Σ[(*PX* × (1 − *PY*)) + ((1 − *PX*) × *PY*)]/*n* between the two species, separately for allopatric and sympatric samples: *P* represents the frequency of a focal allele in the sample of species *X* and *Y*, and *n* is the sequence length (Cruickshank & Hahn, 2014; Nei & Li, 1979). Note that this difference in *d*_*xy*_ between populations is analogous to the *D* statistic (ABBA‐BABA test) for unrooted samples; that is, in the absence of gene flow, we expect equal *d*_*xy*_ for sympatric and allopatric comparisons. The analysis excluded four individuals in the Berwickshire sample identified as obvious early‐generation hybrids or backcrosses (see Section 2.1) and therefore involved 12 allopatric *G. rivale*, 20 allopatric *G. urbanum*, 48 sympatric *G. rivale* and 45 sympatric *G. urbanum* samples. We estimated *d*_*xy*_ for each scaffold (*n* = 418 scaffolds), which we assume to be independent with respect to linkage, and determined the mean *d*_*xy*_ and its *SE* across scaffolds. All analysed radtags were present in at least 12 individuals for each species/population type combination.

As a point of comparison for these two *d*_*xy*_ estimates, we similarly calculated *d*_*xy*_ between the two most diverged allopatric UK *G. rivale* populations (Ben Lawers and Coire Garblach; see Section 2.1). This calculation used all four individuals sampled from each population.

### 2.5 Genetic analysis of hybrids and introgression in sympatric populations

#### 2.5.1 Cluster analysis using faststructure

As a first approach to analysing introgression between the two *Geum* taxa in sympatry, we used genotypic clustering implemented in faststructure (Raj, Stephens, & Pritchard, 2014). Our analysis assumed that samples derived from two populations (i.e., *K* = 2) corresponding to the two *Geum* species, with the possibility of genetic admixture of individuals. The SNP data were derived from a stacks analysis of all 96 successfully genotyped individuals in the sympatric Berwickshire sample together with data from the British and European allopatric populations of the two species. We analysed two differently filtered data sets: (i) SNPs were callable in any fraction of individuals, which retained more SNPs but included missing values for some individuals, and (ii) all SNPs were required to be callable in all individuals. Both analyses used SNPs that had been filtered for paralogs, and considered only a single SNP per scaffold to minimize the effect of linkage between SNPs.

#### 2.5.2 Identifying hybrids and introgressed individuals using species‐specific SNPs

In our second approach to analysing recent introgression, we used custom scripts and the species‐specific SNPs identified in (Section 2.3.4) (above) to estimate the fraction of alleles in each individual within the sympatric Berwickshire sample that is *G. rivale* in origin (Hybrid Index, HI). SNPs with either uncallable genotypes (based on stacks’ likelihood algorithm) or third alleles (e.g., due to sequencing or mapping error) were excluded. We tabulated the frequency of SNPs that were heterozygous for the species‐diagnostic alleles in each individual.

In general, our analyses did not specify minimum coverage because stacks accounts for coverage when calling genotypes (Catchen et al., 2013; Hohenlohe et al., 2010). However, to check whether this could affect our results, we repeated our analyses, requiring a minimum coverage of 20 in all analysed individuals when running faststructure and when creating the panel of species‐diagnostic SNPs. This quartered the number of species‐diagnostic SNPs, but yielded qualitatively identical results (not shown).

### 2.6 Analysis of morphological variation in sympatric populations

#### 2.6.1 Measurement of morphological variation in sympatric populations

From April 2014, cultivated plants from sympatric populations were monitored weekly for flowering. From each plant, a newly opened flower and the stipule on the flowering stem immediately below the flower were sampled. The following characters, which discriminate between *G. rivale* and *G. urbanum*, were measured: angle at which flowers are held (degrees from vertical) (FA), petal length (mm), petal width (mm), petal shape (proportional height of widest part of petal), sepal length (mm), stamen number, stipule length (mm) and stipule width (mm) (Ruhsam et al., 2011, 2013).

#### 2.6.2 Statistical analysis of morphological data

Principal component analysis (PCA) was conducted on the total sympatric sample using data on all eight morphological characters. Distinct groupings were recognized on scatterplots of the first two principal component scores and related to parental species and hybrid classes defined genetically by species‐specific SNPs. PCA based on the same characters was also used to summarize morphological variation separately within *G. urbanum*. GLMs were used to test for the effect of two SNP variants, putatively jointly introgressed from *G. rivale* (see Section 2.1), on the first two principal component scores for *G. urbanum*. All statistical analyses were conducted in minitab 16.

We further characterized two putatively introgress scaffolds that correlated with morphology (see Section 2.1) by measuring *d*_*xy*_ in two ways. For the five radtags found on these scaffolds, we calculated *d*_*xy*_ (as above) between the seven Berwickshire *G. urbanum* samples that putatively possessed these sequences due to introgression vs. either (i) Berwickshire *G. rivale* samples or (ii) the remaining Berwickshire *G. urbanum* samples. Both *d*_*xy*_ calculations excluded four obvious early‐generation hybrids (see Section 2.1).

## 3 RESULTS

### 3.1 Development of SNP markers via ddRAD

#### 3.1.1 Draft genome

The genome assembly contained a total of 170,030 scaffolds, with an N50 of 24.6 kb and total assembly size of 1.2 Gb. To identify signs that scaffolds represent multiple copies of the genome, the distributions of coverage per scaffold and per cent variant bases per scaffold was assessed. Scaffolds of length <10 kb were excluded, leaving 32,182 scaffolds (with a total length of 909 Mb). The distribution of per cent variant bases per scaffold is shown in Figure S2, and the distribution of coverage per scaffold is shown in Figure S3. Coverage per scaffold follows a roughly normal distribution, which would be expected if the scaffolds mostly represented the same number of copies of the genome.

Core genic regions were well assembled and appeared to be present in approximately three (haploid) copies as expected for an ancient hexaploid. We searched our assembled genome for 248 ultra‐conserved eukaryotic genes (CEGs), listed by Parra, Bradnam, and Korf (2007), using their Core Eukaryotic Genes Mapping Approach. We identified 93% and 97% of the core genes that were complete or partially complete, respectively. On average, complete and partially complete CEGs were represented by 3.39 and 3.82 orthologs per CEG, respectively, with at least 90% of CEGs represented by more than one ortholog.

#### 3.1.2 ddRAD tags

Following quality filtering, 2.7 × 10^7^ reads remained in the miseq data, and between 6.0 × 10^7^ and 7.3 × 10^7^ reads remained that derived from the five hiseq libraries. Following alignments, assembling radtags with stacks and applying corrections with rxstacks, our data set included 230,356 radtags for *G. rivale* and *G. urbanum*, collectively. However, coverage was highly stochastic. For example, only *c*. 2% (4,524) of radtags were represented in more than one half of our samples.

#### 3.1.3 Identifying and filtering for paralogs

When the stacks’ populations module was used to analyse the raw SNP data from the allopatric populations of the inbreeding taxon *G. urbanum*,* F*_IS_ estimates were low and sometimes negative, which is unexpected for a highly inbreeding species. This suggests that paralogous reads have mapped to identical locations and thereby increased individual heterozygosity (see Table S4). We therefore applied our filter for paralogy (rejecting 1,344 scaffolds with SNPs that exhibit either excess heterozygosity (>0.5) or negative *F*_IS_) and, unless specifically noted, we henceforth only present results from paralogy‐filtered SNPs.

### 3.2 Population genetic analysis of allopatric populations

#### 3.2.1 Patterns of polymorphism within and between species

The majority of SNPs in the data set were polymorphic in *G. rivale* but invariant in *G. urbanum*. This type of polymorphic site occurred approximately four times more frequently than the reverse case (Table 1). 22% of SNPs were alternately fixed between the species (Table 1) and only 1.5% of SNPs were shared (Table 1).

**Table 1:** Frequency of polymorphism of different forms within and between *Geum* species

Polymorphism type	Count
Alternately fixed SNPs	488
SNP polymorphic in *Geum rivale* but one allele fixed in *Geum urbanum* (e.g., *G. rivale*: A, T; *G. urbanum*: T)	1,334
SNP polymorphic in *G. urbanum* but one allele fixed in *G. rivale*	338
*G. rivale* and *G. urbanum* share polymorphism	34
Total SNPs analysed	2,194

Analysis allows multiple SNPs per scaffold.

#### 3.2.2 Inbreeding coefficients and population differentiation

For the allopatric populations sampled in Britain, we obtained *F*_IS_ estimates close to 0.25 for *G. rivale*, consistent with leaky self‐incompatibility (Ruhsam et al., 2010), and *F*_IS_ > 0.9, consistent with very high selfing rates, for eight of the 10 UK “allopatric” *G. urbanum* populations (Table 2). Among the two *G. urbanum* populations with low *F*_IS_, the first population (Mill Wood *F*_IS_ = 0.0845), included one (of the two) sample(s) that was heterozygous at 92% of the 71 polymorphic SNPs analysed, suggesting that this sample was derived from a recent outcrossing event between two different inbred lines. In the second population, Selwyn Wood, only one SNP was recorded as polymorphic, suggesting that this population may have been founded by few (possibly a single) highly selfing individuals.

**Table 2:** *F*_IS_ estimates for “allopatric” UK *Geum rivale* and *Geum urbanum* populations using data filtered for paralogs

Population by species	Number of SNPs	*F*_IS_
*G. rivale* (*n* = 4 for all populations)		
Ben Lawers	309	0.24
Coire Garblach	347	0.26
Ben Lui	341	0.24
*G. urbanum* (*n* = 2 for all populations)		
Priory Wood	67	0.98
Mill Wood	71	0.08
Burgh Wood	76	0.89
Hoades Wood	47	0.95
Punnetts Town	61	0.85
Frith Wood	69	0.96
Stanford Bridge	68	0.97
Combe Wood	62	0.99
Copperhurst	67	0.99
Selwyn Wood	Only 1 polymorphic locus	

Allopatric populations of *G. rivale* in Britain exhibited less population differentiation than *G. urbanum* populations (mean pairwise *F*_ST_ 0.13 and 0.38, respectively), as expected from their contrasting mating systems.

#### 3.2.3 Coalescent analysis of gene flow during lineage divergence

Table S5 summarizes the numbers of each polymorphism type and blocks analysed for the *G. urbanum*–*G. rivale* sample pairs. All three pairs had approximately 1,400 SNPs distributed among *c*. 660 blocks (i.e., radtags). Model comparisons for all three sample pairs reject a model of strict divergence and suggest that introgression has occurred between the *Geum* species (Table 3), but at a very low rate (see below). For two sample pairs (involving *G. rivale* samples Ben Lui 1 and Ben Lawers 5), the model of gene flow from *G. rivale* to *G. urbanum* (i.e., IM_r➔u_) fits the data significantly better than the model of strict divergence (i.e., div_2_), whereas the model IM_u➔r_ does not fit significantly better than div_2_ (Table 3). Models that include gene flow (IM_r➔u_ and IM_u➔r_) also fit the data significantly better than the div_2_ model for the third pair (involving sample Ben Lui 4), but IM_r➔u_ and IM_u➔r_ have effectively equal support (Table 3). Results from this latter pair likely differ from the former pairs because it includes a single block with a shared heterozygous site, while the other sample pairs lack shared heterozygous sites (Tables S5 and S6). As Ben Lui 4 is likely the most heterozygous *G. rivale* sample (Table S5), we focus on this pair, but note that parameter estimates (Table S6) and general conclusions (i.e., support for very low introgression rates) are similar for all three sample pairs.

**Table 3:** Difference in log Likelihoods between the “best” (log Likelihood = 0) and alternate models; models with difference <−2 fit data significantly worse than the best model

Sample	div_2_	IM_u→r_	IM_r→u_
Ben Lui 1	−12.9	−12.5	0
Ben Lui 4	−9.66	0	−0.721
Ben Lawers 5	−6.67	−6.51	0

“Sample” refers to which *Geum rivale* individual is paired with the single *Geum urbanum* individual. div_2_: species diverged with no introgression; IM_u→r_: divergence with gene flow from *G. urbanum* to *G. rivale*; IM_r→u_: divergence with gene flow from *G. rivale* to *G. urbanum*.

The three models (div_2_, IM_r➔u,_ IM_u➔r_) yield similar estimates of *N*_e_ and divergence time (Ben Lui 4 pair: Table 4; all three sample pairs: Table S6). In general, *N*_e_ of *G. urbanum* is a half to a quarter of that of *G. rivale* (and their common ancestor), and all three models suggest that the species diverged approximately 2–3 Mya (Table 4; Table S6). Models IM_u➔r_ and IM_r➔u_ both suggest a low but significant long‐term rate of effective gene flow (*M* ≈ 0.04), of approximately one migrant every 25 generations (Table 4; see Table S6 for *M* for additional sample pairs).

**Table 4:** Parameter estimates under the models for *Geum rivale* sample, Ben Lui 4. 95% Confidence intervals provided in parentheses

	Model		
	div_2_	IM_r→u_	IM_u→r_
*N*_anc_ = *N*_riv_	1.30 × 10^5^	1.24 × 10^5^	1.11 × 10^5^
*N*_urb_	3.77 × 10^4^	3.09 × 10^4^	3.38 × 10^4^
*t* (years)	2.22 × 10^6^ (2.01 × 10^6^–2.43 × 10^6^)	2.35 × 10^6^	2.48 × 10^6^
*M* (mean, 95% CI's)		0.04 (0.005–0.191)	0.04 (0.007–0.101)

See Table S6 for parameter estimates for all three sample pairs. *N*_anc_, *N*_rivb*,*_ *N*_urb_: Effective population size of common ancestor, *G. rivale* and *Geum urbanum*, respectively. *t*: years since species divergence. *M* = 4*N*_anc_ × *m* individuals per generation (*m* = probability of migration each generation). See Table 3 or text for model definitions.

### 3.3 Population genetic comparison of allopatric and sympatric populations

#### 3.3.1 f3 tests for introgression

f3 tests provided no evidence for long‐term introgression from UK allopatric *Geum* populations into either *G. rivale* or *G. urbanum* Berwickshire populations. Mean f3 was negative (−0.00016) when calculated for Berwickshire *G. rivale* samples, but did not differ significantly from zero (95% CI's: −0.00168 to 0.00157; based on 3,945 SNPs across 493 scaffolds). f3 was positive (0.00174) for the Berwickshire *G. urbanum* samples and again did not differ significantly from zero (95% CI's of −0.00055 to 0.00397; based on 2,751 SNPs across 484 scaffolds).

#### 3.3.2 Genetic differentiation between taxa in allopatry and sympatry

Genetic differentiation between *G. rivale* and *G. urbanum* is similar for UK allopatric and sympatric samples (mean *d*_*xy*_ (± *SE*) equals 0.0115 ± .0005 and 0.0112 ± .0005, respectively). By comparison, *d*_*xy*_ between the two most diverged allopatric UK *G. rivale* populations equalled 0.00286 ± .00008. We note that, due to the fact that stacks ignores monomorphic radtags, these estimates should be viewed as relative measures (and upper limits) of *d*_*xy*_, and not as absolute estimates (see also Arnold, Corbett‐Detig, Hartl, & Bomblies, 2013).

### 3.4 Hybrids and introgression in sympatric populations

#### 3.4.1 Cluster analysis using faststructure

**Figure 2:**
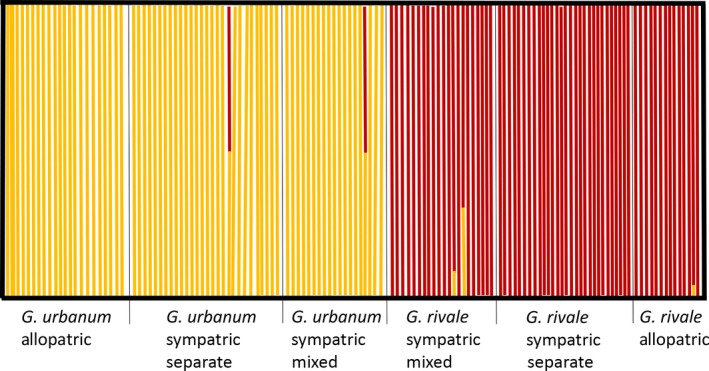
Results of faststructure (Raj et al., 2014) cluster analysis for *K* = 2 based on 188 SNP loci present in all 133 *Geum* individuals scored in the allopatric and sympatric populations. Each bar represents one individual and shows the proportion of the genome from *Geum rivale* (red) and *Geum urbanum* (yellow) [Colour figure can be viewed at http://wileyonlinelibrary.com]

faststructure analyses that considered SNPs present in either all (188 SNPs) or a fraction of (492 SNPs; results not shown) individuals analysed in the combined allopatric and sympatric populations yielded highly consistent results. Figure 2 illustrates faststructure results for analyses based on 188 SNPs. All except one of the 36 individuals in the allopatric populations, and all except four of the 96 individuals in the sympatric populations, show <1% admixture. In the sympatric population, *Q* values (proportion of *G. rivale* genome) of the four individuals with substantial admixture are 0.496, 0.517, 0.663 and 0.935. These *Q* values provide no evidence for significant, advanced introgression. The substantially admixed individuals present are likely recently formed F1 or F2 hybrids, and different generations of backcrosses to *G. rivale*.

#### 3.4.2 Identifying hybrids and introgressed individuals using species‐specific SNPs

**Figure 3:**
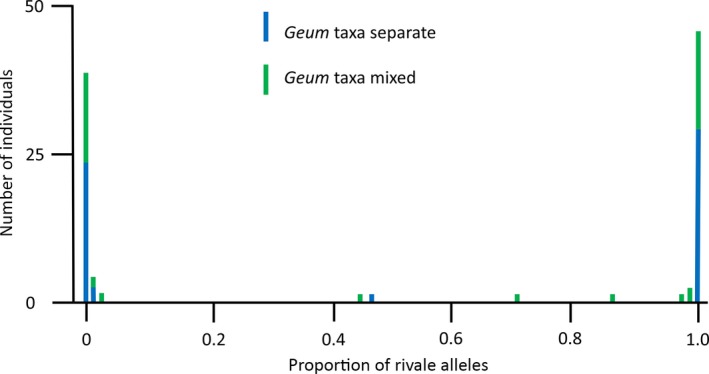
Distribution of hybrid index (proportion of *Geum rivale* species‐specific alleles) for 96 individuals within 39 sympatric populations where *G. rivale* and *G. urbanum* were either found alone (blue bars, “pure population”) or together (green bars, “mixed population”). Mean number of loci analysed per individual = 207.3 [Colour figure can be viewed at http://wileyonlinelibrary.com]

To initially assess the ability of the species‐specific SNPs to discriminate the species, we genotyped the 10 British allopatric *G. urbanum* samples that had not been used to generate species‐diagnostic SNPs; 215 of 220 diagnostic SNPs were homozygous for the *G. urbanum* allele in all individuals. Five SNPs were homozygous for the putative *G. rivale* allele in one or more of the 10 *G. urbanum* individuals, indicating that these SNPs were not fixed between species. They were removed from subsequent analysis of 96 individuals of *Geum* from the Berwickshire population. The mean number of SNPs successfully scored per individual was 207.3 (range 161–215; Figure S5). Figure 3 illustrates variation in HI in the Berwickshire population.

The vast majority of individuals in the total sympatric sample (92 of 96) have genotypes in which more than 97% of the species‐specific SNPs derive from only one of the two *Geum* species. Individuals possessing >97% *G. rivale* variants are hereafter regarded as *G. rivale*, while plants with >97% *G. urbanum* variants are classified as *G. urbanum*. The four remaining individuals with substantial proportions of variants from both species correspond to the four early‐generation hybrids identified by the faststructure analysis. The *Q* values calculated by faststructure and the hybrid index calculated here are highly correlated (see Figure S6 for comparison using 188 SNPs), in part because some SNPs (i.e., 61 of the 492 SNPs available for faststructure analysis) are common to both analyses. Early‐generation hybrids were significantly associated with “mixed” rather than “pure” sites within Berwickshire (Fisher's exact test, *p* = .044). We highlight, however, the lack of evidence for introgression even in the 10 sites that had both species present at the time of sampling: the vast majority of these 40 samples (10 sites × 4 individuals per site; see Section 2) had >97% of species‐specific alleles from one species.

Neither of the two individuals possessing a hybrid index close to 0.5 are heterozygous at all SNPs, the situation anticipated if they were simple F1 hybrids (Table 5). Instead, they are homozygous at a substantial fraction of the species‐specific SNPs (15% and 18%), suggesting that they may have been derived by selfing of F1 hybrids. This is supported by the observation that, where additional species‐specific markers occurred on the same scaffold as the originally scored homozygous SNP (*n* = 9, *n* = 12 respectively), they were also homozygous for alleles specific to the same taxon. Two further individuals contain alleles derived predominantly from *G. rivale*, but additionally possess a substantial fraction of *G. urbanum* alleles (29% and 12%, respectively; Table 5). On the basis of their complement of alleles alone, these plants most likely represent first‐ and second‐generation backcrosses to *G. rivale,* respectively. However, their origins must again be more complex, possibly involving selfing, because a substantial fraction of the *G. urbanum* specific alleles they possess (10% and 33%, respectively) are present in homozygous form. All other *G. urbanum*‐specific alleles (*n* = 2, *n* = 3, respectively) present on the same scaffolds were also homozygous. If these hybrids had been simple backcrosses, all *G. urbanum* alleles would have been present as heterozygotes.

**Table 5:** Genotypic composition at species‐specific SNPs for individuals classified as early‐generation hybrids (H) and backcrosses to *Geum rivale* (BCR)

Sample and classification	% “*rivale*” in genome	Homozygous “*rivale*” allele	Heterozygous	Homozygous “*urbanum*” allele	Total Loci scored
29A (H)	47.2	9	166	20	195
32C (H)	45.3	7	132	22	161
26B (BCR)	87.9	138	28	7	173
27B (BCR)	70.8	95	113	6	214

Among the 92 plants from Berwickshire possessing a preponderance (>97%) of species‐specific alleles from one taxon, 33 of the 47 individuals assigned to *G. rivale* and 26 of the 45 plants assigned to *G. urbanum* possessed from 1 to 10 alleles classified as specific to the alternate species (Figure S7). These could represent alleles that have introgressed, or alternatively alleles present at low frequency in the focal species that have not been detected in the limited sample of allopatric genotypes used to identify species‐specific SNPs. The maximum frequencies of putatively introgressed alleles are low (9.6% and 14.4% in *G. rivale* and *G. urbanum,* respectively). Furthermore, 76% are polymorphic in *G. rivale* only, 20% are polymorphic in *G. urbanum* only, and 4% are polymorphic in both species. These proportions are not significantly different from those found in the total sample of SNPs scored (*p* > .05, Table 1). Therefore, misclassification of species‐specific SNPs likely explains the apparent presence of up to 3% admixture in the *Geum* genomes from sympatric populations, rather than introgression.

With one exception, the putatively introgressed alleles discussed above were randomly associated with each other within each of the sympatric *G. rivale* and *G. urbanum* populations. However, two species‐diagnostic SNPs at radtags 20,454 and 24,791, respectively, and originally classified as specific to *G. rivale*, showed complete association in genotypic state within sympatric *G. urbanum*. The alleles were present at a mean frequency of 14.4%, distributed across six sites. In six individuals, both alleles were present in homozygous form, while a seventh individual was heterozygous at both SNPs. As the SNPs involved are located on different scaffolds, these results may indicate the presence of a section of genome spanning the two scaffolds, possibly derived from *G. rivale*, which is segregating in the *G. urbanum* population. When two additional species‐specific SNPs located on the scaffold, but up to 19.5 kb from radtag 24,791, were analysed, these were also found to possess alleles specific to *G. rivale* and to be in complete linkage disequilibrium with the original SNP scored. This provides further evidence for a large section of un‐recombined DNA, derived from *G. rivale*, segregating in the sympatric *G. urbanum* population.

The *d*_*xy*_ value calculated for the putatively introgressed *G. rivale* scaffolds also supported this hypothesis, as these *d*_*xy*_ estimates mirrored the mean between‐species *d*_*xy*_ estimates based on all radtags. The putatively introgressed scaffolds in *G. urbanum* showed little divergence from Berwickshire *G. rivale* samples (mean *d*_*xy*_ = 0.00287); this divergence was very similar to that observed between allopatric *G. rivale* populations (see Section 2.1, above). By contrast, we observed higher divergence between the putatively introgressed scaffolds in the seven focal *G. urbanum* samples and the remaining *G. urbanum* Berwickshire samples (*d*_*xy*_ = 0.01323); this *d*_*xy*_ estimate was similar to that observed between species, when calculated for all radtags (see Section 2.1, above).

### 3.5 Analysis of morphological variation in sympatric populations

#### 3.5.1 Correspondence between phenotypic and genotypic classification

**Figure 4:**
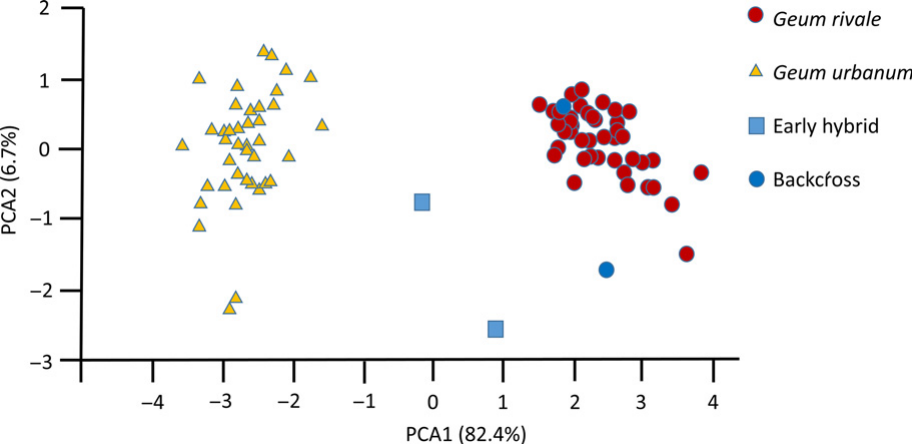
Principal component analysis analysis based on eight morphological characters measured in a common environment for a sample of 87 individuals of *Geum* sampled from sympatric populations. Genetic class (from Hybrid Index) of each individual (*G. rivale*,* G. urbanum*, early hybrid, or backcross to *G. rivale*) derived from an analysis of 215 species‐specific markers is shown [Colour figure can be viewed at http://wileyonlinelibrary.com]

Morphological measurements of eight floral and vegetative characters were obtained from 87 of the 96 individuals from Berwickshire scored for species‐specific SNPs. Based on these data, PCA1 and PCA2 accounted for 82.4% and 6.7% of the variation, respectively, and distinguished two major groups, with two individuals falling between these on the first PCA axis (Figure 4). Individuals classified as *G. rivale* (*N* = 43) and *G. urbanum* (*N* = 40) on the basis of species‐specific SNPs fall clearly into the two groups with positive and negative PCA1 values, respectively. The two individuals with intermediate PCA1 scores correspond to the early‐generation hybrids identified with clustering and species‐specific SNPs. The two genotypes identified as complex backcrosses to *G. rivale*, on the basis of species‐specific SNPs, group morphologically with individuals classified as *G. rivale* on the PCA plot.

#### 3.5.2 Morphological variation and putative introgression

**Figure 5:**
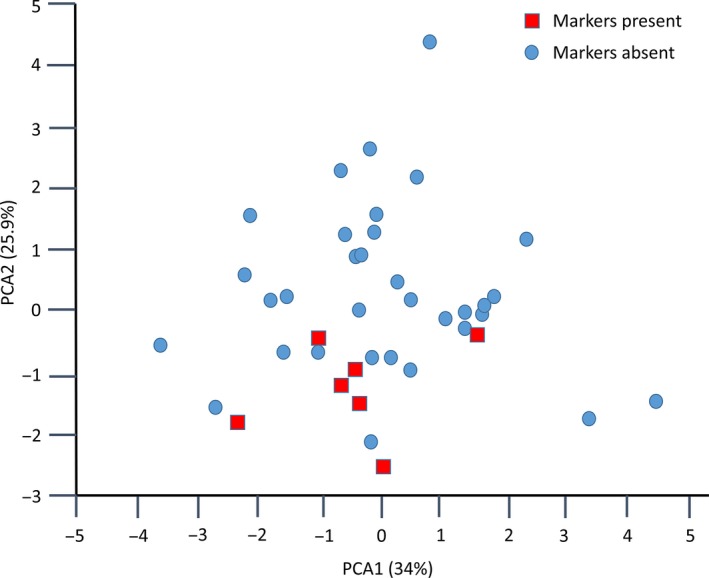
Principal component analysis analysis of 40 individuals of *Geum urbanum* sampled from sympatric populations based on variation for eight morphological characters measured in a common environment. Red, square individuals jointly possess species‐specific SNPs from *Geum rivale* at radtags 20,454 and 24,791 [Colour figure can be viewed at http://wileyonlinelibrary.com]

We used PCA generated, above, to determine whether presence of putatively introgressed genetic material marked jointly by alleles at radtags 20,454 and 24,791 affected the morphology of *G. urbanum* in Berwickshire. Analysis of variance showed no significant effect of joint presence/absence of the alleles on PCA1, but a significant effect on PCA2 score (*F*_1,38_ = 8.67, *p* = .005; Figure 5). Individuals of *G. urbanum* jointly possessing these alleles showed a significantly larger flower angle than those lacking them (91.4 vs. 60.8 degrees, *p* = .018). Greater flower angle is a feature characteristic of *G. rivale* and is most heavily weighted in PCA2.

## 4 DISCUSSION

Our comparative genomic analyses of allopatric and sympatric populations of *G. rivale* and *G. urbanum* have allowed us to measure historical and contemporary gene exchange in this outcrossing and selfing species pair. Coalescent modelling of RAD data from allopatric populations suggests that the sister species split 2–3 Mya. Although we find some support for models that include gene exchange between the species between the time they split and the isolation of the allopatric populations, the estimated long‐term rate of introgression is extremely low (1 genome per 25 generations); also, violation of model assumptions could yield a spurious signal of introgression (see below). Similarly, neither a comparison of genetic differentiation between allopatric and sympatric populations nor an f3 test provides evidence for significant recent introgression within an area of sympatry. This conclusion agrees with both clustering analyses and analysis of species‐specific markers, each suggesting little (if any) admixture in Berwickshire. Thus, our analyses suggest that, despite widespread contemporary hybridization, introgression between *G. rivale* and *G. urbanum* has not been important in their evolution. The only piece of evidence that contradicts this general conclusion is the detection of a >19.5 kb DNA sequence, apparently derived from *G. rivale*, segregating within the sympatric *G. urbanum* population. Although this introgressed genetic material significantly affects the morphology of *G. urbanum plants*, it is present at a frequency of 0.14 in Berwickshire *G. urbanum* population and involved a small fraction of the *Geum* genome (<1% of our species‐diagnostic SNPs).

Our coalescent analyses suggest that the *Geum* species are considerably older (2–3 Myr) than estimates for other sister taxa that possess contrasting outcrossing and selfing mating systems. These range from 50 to 100 kya for *Capsella grandiflora* and *C. rubella* (Brandvain, Slotte, Hazzori, Wright, & Coop, 2013) through 500–1,100 kya for *Mimulus guttatus* and *M. nasutus* (Aeschbacher, Selby, Willis, & Coop, 2016) to 1 Mya for *Arabidopsis lyrata* and *A. thaliana* (Tang et al., 2007). Our estimated age of the *Geum* species is consistent with the numerous morphological and physiological differences that are exhibited between them (Taylor, 1997a, 1997b). However, despite their age, *G. rivale* and *G. urbanum* apparently exhibit few intrinsic hybrid incompatibilities (Ruhsam et al., 2013); these observations parallel those from *Drosophila* species, which show few postzygotic incompatibilities for species of a similar age (Coyne & Orr, 1989, 1997), but are in contrast to what has been reported for a number of other sister species of plants (see Brennan, Hiscock, & Abbott, 2014).

Coalescent analyses also suggest that very low levels of introgression may have occurred between the *Geum* species up to the time of isolation of allopatric populations, with no strong signal of introgression in a particular direction. For clarity, *M* (= 4*N*_anc_ *m*; see Section 2) measures long‐term effective migration and accounts for reduced migration due to selection against hybrids (see below). Our estimated ancient introgression rate between the *Geum* species (*M* = 0.04 diploid genomes per generation) is an order of magnitude lower than that from other inbreeding/outbreeding pairs: *M. nasutus* into *M. guttatus* (*M* = 0.1–1; Aeschbacher et al., 2016); between subspecies of *Clarkia* in their secondary contact zone (*M* = 0.897–0.169; Pettergill & Moeller, 2012); from *Oryza nivaria* to *O. rufipogon* (contemporary gene migration, *M* = 0.23; Zhou et al., 2008).

Our coalescent analyses necessarily rely on simplifying assumptions. First, the blockwise site‐frequency spectrum approach assumes a constant *N*_e_ and μ across blocks and so ignores effects of background selection on linked sites and heterogeneity in mutation rate, which could lead to a spurious signal of introgression (Ewing & Jensen, 2016). Indeed, this coalescent method has predicted a smaller proportion of monomorphic blocks than observed in the data set, both in previous analyses (e.g., Nürnberger, Lohse, Fijarczyk, Szymura, & Blaxter, 2016) and for our own data (not shown). Such under‐prediction likely arises because the model assumes neutrality, when selective constraints likely maintain some blocks as monomorphic. Second, our models represent drastic simplifications of these species’ histories; in particular, they assume constant *N*_e_ in *G. rivale* and the ancestral population, which is unrealistic given the climatic fluctuations and range shifts of European taxa during the Pleistocene. Following species divergence, either a change in *N*_e_ or introgression can alter the joint site‐frequency spectrum (Chen, 2012); by assuming that population size of the ancestral population equals that of *G. rivale* (*N*_anc_ = *N*_riv_), we potentially conflate changes in *N*_e_ with introgression, which, again, could lead to a spurious signal of introgression. Given these issues and the extremely low estimates of introgression, we do not interpret support of IM models over the div2 model as strong evidence for ancient long‐term gene flow between the *Geum* species (i.e., *M* could equal 0).

We also found little evidence for recent introgression. Our first tests of introgression in contemporary populations involved comparisons of allopatric vs. sympatric populations: we did not find reduced species differentiation (*d*_*xy*_) in sympatry compared with allopatry, nor any signal of introgression in the f3 analysis (Patterson et al., 2012). As a second approach to identify recent introgression, we compared the genomic composition of individuals in sympatry (Berwickshire) to samples from allopatric, reference populations. Both the faststructure analysis (which did not rely exclusively on species‐diagnostic SNPs) and the hybrid index based on species‐diagnostic SNPs identified the same four individuals as early hybrids and backcrosses; they yielded highly correlated (*r* = .99) estimates of genomic composition, and both approaches matched morphological classifications. Leaving aside the four early‐generation hybrids identified, our analyses suggest that 35% of the Berwickshire samples scored showed no detectable introgression. Within the remaining samples, the upper limit for the proportion of introgressed genome is between 1% (cluster analysis) and 3% (species‐specific markers). At the least, the 3% estimate is likely to be inflated because the assumption that all our species diagnostic SNPs are alternately fixed between the species is unlikely to be true. These estimates apply to a broad “sympatric” region, including 10 sites where the *Geum* species co‐occurred and 29 sites where they were found individually at the time of sampling.

Although our overall results indicate very little recent genetic exchange, one intriguing result provides tentative evidence for very limited introgression from *G. rivale* to *G. urbanum*. Four SNPs specific to *G. rivale* in alloparic populations, located on two scaffolds covering more than 19.5 kb, cosegregated within the sympatric *G. urbanum* population. These scaffolds also showed lower differentiation from equivalent scaffolds in *G. rivale* than from those in *G. urbanum*. Moreover, *G. urbanum* individuals possessing these alleles exhibited a significantly greater flower angle, characteristic of *G. rivale*. This may represent partial introgression of a chromosome segment from *G. rivale* into *G. urbanum*, currently at a low frequency within the sympatric population. Given the absence of evidence for backcrossing of hybrids to *G. urbanum* (Ruhsam et al., 2011, 2013), such introgression could only arise through the establishment, following hybridization, of a self‐fertilizing lineage that largely comprises genetic material from *G. urbanum*, but contains this section of chromosome from *G. rivale*. Previous work revealed that F1 hybrids are self‐compatible and a proportion may be capable of autopollination, so this scenario is not impossible (Ruhsam et al., 2013). While such putative introgression seems to have contributed novel genetic diversity to the inbreeding species, any evolutionary impact may be limited due to the low frequency of the introgressed material (0.14). Moreover, its presence in the *G. urbanum* population may be temporary.

Despite the reproductive barrier that a selfing mating system naturally imposes (Briscoe Runquist, Chu, Iverson, Kopp, & Moeller, 2014; Brys, et al., 2014; Martin & Willis, 2007), the widespread occurrence of the F1 hybrids throughout the area of sympatry of *G. rivale* and *G. urbanum* in Berwickshire and elsewhere (Preston et al., 2002), and our own detection of early‐generation hybrids, indicates that low levels of introgression are not caused by lack of opportunity for hybridization. Previous studies show that where hybridization occurs, backcrossing with *G. rivale* and self‐fertilization yields a wide array of fertile recombinant offspring, which show little evidence of intrinsic genetic incompatibility under benign conditions (Ruhsam et al., 2011, 2013). Thus, *G. rivale* and *G. urbanum* join a growing list of sister species that readily produce F1 hybrids and viable early recombinant offspring, yet show little or no signal of contemporary introgression beyond this stage (e.g., *Encelia* (Kyhos, Clark, & Thompson, 1981); *Quercus* (Nason, Ellstrand, & Arnold, 1992); *Rhododendron* (Milne, Terzioglu, & Abbott, 2003); *Begoni*a (Twyford, Kidner, & Ennos, 2015; F1s mainly sterile); *Populus* (Christe et al., 2016); *Bombina* (Nürnberger et al., 2016); see Abbott (2017) for further details and examples).

In many of the above examples, ecological selection against recombinants has been invoked to explain the maintenance of distinct species; this may also account for the paucity of introgression in *Geum*. Since their initial divergence, the *Geum* species have evolved marked differences in pollination syndrome, phenology, vegetative morphology and many aspects of physiology including tolerance of shade and waterlogging (Taylor, 1997a, 1997b; Waldren, Etherington, & Davies, 1988). Therefore, multiple ecologically important characters could potentially be the targets of selection against recombinant individuals in natural populations (Nosil, Harmon, & Seehausen, 2009). Selection may be particularly effective in the self‐fertilizing recombinants that are required to facilitate introgression from *G. rivale* into *G. urbanum*. Here, reduced effective recombination rates will lead to correlated selective effects across the genome, and deleterious recessive mutations present as genetic load in genome segments derived from outcrossing *G. rivale* will also be exposed (Hu, 2015). Given our ability to artificially cross the *Geum* species and produce large numbers of later generation hybrids, there is now the potential to test experimentally the hypothesis that strong ecological selection against recombinant genotypes maintains contemporary species integrity.

## DATA ACCESSIBILITY

Draft genome of G. urbanum available from the European Nucleotide Archive (ENA): http://www.ebi.ac.uk/ena/data/view/PRJEB23412

DNA sequences:

Read used to produce draft *G. urbanum* genome available the European Nucleotide Archive (ENA): http://www.ebi.ac.uk/ena/data/view/PRJEB23267.

ddRAD reads also available from ENA: http://www.ebi.ac.uk/ena/data/view/PRJEB23012.

Morphological data available on Dryad: https://doi.org/10.5061/dryad.k67fv.

Scripts available on Github, at crispinjordan/Geum‐introgression.

## CONFLICT OF INTEREST

None declared.

## AUTHOR CONTRIBUTIONS

C.Y.J., K.G. and R.A.E. conceived the study; C.Y.J., F.T., M.T. and R.A.E. gathered data; C.Y.J., K.L., F.T. and R.A.E. analysed data; C.Y.J., K.L. and R.A.E. wrote the manuscript with input and revisions from all co‐authors.
