# Supplementary material for: Maintaining their genetic distance: Little evidence for introgression between widely hybridizing species of Geum with contrasting mating systems
Source: Mol Ecol. 2017 Dec 9;27(5):1214–28. doi: 10.1111/mec.14426 (PMC5900869; doi:10.1111/mec.14426)
Supplement: Supplementary file 1 [file MEC-27-1214-s001.pdf]

SUPPLEMENTARY MATERIALS

Maintaining their genetic distance: little evidence for introgression between widely hybridising species of *Geum* with contrasting mating systems

Crispin Y. Jordan[1,3,4], Konrad Lohse[1], Frances Turner[2], Marian Thomson[2], Karim Gharbi[2], Richard A. Ennos[1]

SUPPLEMENTARY MATERIAL

i. Development of Draft Genome for *G. urbanum*

Reads were generated from three Illumina libraries: a 151 paired end library with a mean insert size of 400 (sequenced on Hi seq 2500 (v1 chemistry) to give 144 million read pairs), a 151 paired end library with a mean insert size of 600 (sequenced on Hi seq to give 134 million read pairs), and a 251 paired end library with a mean insert size of 400 (sequenced on Illumina Mi seq (v2 chemistry) to give 11.9 million read pairs). Illumina adapters were removed and reads quality trimmed using cutadapt version 1.3 (Marcel, 2011) with the option "-q 30". Trimmed reads were assembled into contigs and scaffolds using MaSuRCA version 2.0.3.1 (Zimin et al., 2013). Following the user guide's recommendations, we left all options as default, except that we set cgwErrorRate=0.25 and JF_SIZE=23000000000 (i.e., we set jellyfish hash size to about 10X the genome size, as recommended). Overall, we implemented the options:

GRAPH_KMER_SIZE=auto, USE_LINKING_MATES=1, LIMIT_JUMP_COVERAGE = 60, ovlMerSize=30, cgwErrorRate=0.25, ovlMemory=4GB, KMER_COUNT_THRESHOLD = 1, NUM_THREADS= 64,JF_SIZE=23000000000 , DO_HOMOPOLYMER_TRIM=0.

To identify SNPs within the draft genome and estimate coverage per scaffold, trimmed reads were mapped back to assembled scaffolds using bwa mem version 0.7.5a with -M option (Li & Durbin 2009). 94.0% of reads were aligned to scaffolds. Samtools (Li *et al.* 2009) version 0.1.18 was used to call variants.

ii. Sample Preparation, Library Preparation, and PCR Amplification for ddRAD

For each individual sample, 35ul of genomic DNA (250ung) was digested in a total volume of 50 ul, comprising 1.0 ul SphI-HF (20u), 1.0 ul EcoRI-HF (20u), 5.0 ul 10x Cutsmart buffer (New England Biolabs, UK) and 8.0 ul dH2O for a total of 3 hours, at 37°C. The reaction was purified by the addition of Ampure XP beads at a ratio 1.8 x beads: 1x DNA rather than by heat denaturation, to maintain the integrity of the EcoRI generated overhangs.

Samples were eluted in 25ul EB (Qiagen). Unique barcoded EcoRI P1 adapters (Peterson et al. 2012) and a generic SphI P2 adapter (Table S3) were ligated to the complementary overhangs of the DNA fragments generated by the EcoRI:SphI double digest reaction. Each ligation reaction was carried out in a total volume of 40ul, consisting of 25ul DNA, 2.0 ul P1 adapter (4uM), 2.0 ul P2 adapter (4uM), 4.0 ul 10x T4 Ligase buffer (New England Biolabs, UK) 1ul Quick Ligase (New England Biolabs, UK), and the reaction was incubated at 23°C for 30 min followed by heat denaturation at 65 °C for 15 min.

After ligation the reactions were pooled into libraries containing a maximum of 24 samples, each barcoded with a unique EcoRI PI adapter sequence. The pooled libraries were then subjected to two rounds of Ampure XP bead clean up, at a ratio of 1x Ampure XP beads: 1 x DNA and eluted in a volume of 30ul EB (Qiagen). Individual libraries were size selected by BluePippin Size selection system, 2% Precast Agarose gel cassette (sage science) using the 'Tight' collection mode set to a target size of 300bp. The success of size selection was assessed by Bioanalyzer DNA HS chip (Agilent Technologies).

For each ddRAD library, PCR amplification was carried out in 2 independent 50 µL reactions consisting of 20 ul size selected DNA, 25ul 2× Phusion Master Mix (New England Biolabs, UK), 2.5 ul DMSO, and 1ul ddRAD P1 PCR primer (10 nm), and a barcoded P2 primer (10 nm) that introduced a 8bp unique index during amplification (Table S3), using the following cycling parameters: 98 °C for 30 s followed by 12 cycles of 98 °C for 10 s and 72 °C for 60 s.

PCR products were then pooled, and purified by Ampure XP beads at a ratio of 0.8 x beads: 1x DNA, and the mean insert size of the individual libraries was assessed by Bioanalyzer DNA HS chip, (Agilent Technologies), and were quantified by qPCR on an Illumina Eco instrument using the Kapa Library Quantification Kit.mA final super pool was created consisting of equimolar individual library PCR reactions and the quantification of the pool was measured by qPCR.

References

Marcel M (2012). Cutadapt removes adapter sequences from high-throughput sequencing reads. *Bioinformatics in Action*, **17**, 10-12.

Peterson BK, Weber JN, Kay EH, Fisher HS, Hoekstra HE (2012) Double digest RADseq: an inexpensive method for *de novo* SNP discovery and genotyping in model and non-model species. *PloS one*, **7**, e37135.

Zimin AV, Marçais G, Puiu D, Roberts M, Salzberg SL, Yorke JA (2013)The MaSuRCA genome assembler. *Bioinformatics*, **29**, 2669-2677.

SUPPLEMENTARY TABLES

Site	Coordinates	Taxon
Ben Lawers, UK	56°32’23.46”N/ 004°17’27.58”W	R (4)
Coire Garblach, UK	57°01’38.29”N/ 003°50’08.17”W	R (4)
Ben Lui, UK	56°23’38.87”N/ 004°49’28.02”W	R (4)
Iceland	64°07'N / 21°50'W	R (1)
Sweden	63°17'27.00"N/ 18°42'54.00"E	R (1)
Selwyn Wood, UK	50°57’43.96”N/ 000°12’40.52”E	U (2)
Combe Wood, UK	51°02’05.03”N/ 000°18’31.39”E	U (2)
Burgh Wood, UK	51°01’21.30”N/ 000°27’13.26”E	U (2)
Hoads Wood, UK	51°08’47.89”N/ 000°47’27.02”E	U (2)
Stanford Bridge, UK	51°09’00.56”N/ 000°45’33.55”E	U (2)
Frith Wood, UK	51°10’22.20”N/ 000°42’59.47”E	U (2)
Priory Wood, UK	51°05’07.33”N/ 000°54’02.91”E	U (2)
Copperhurst, UK	51°04’55.62”N/ 000°56’53.11”E	U (2)
Mill Wood, UK	50°58’47.12”N/ 000°24’56.60”E	U (2)
Punnets Town, UK	50°58’20.12”N/ 000°18’43.66”E	U (2)
Portugal	40°22'43.00"N/ 008°22'11.00"E	U (1)
France	43°16'59.57"N/ 001°28'56.59"E	U (1)

Table S1. Sampling locations for allopatric populations of *Geum rivale* (R) and *G. urbanum* (U). Numbers in brackets = sample size.

Site Number	Elevation(m)	Coordinates	Taxa and total number sampled
1	331	55°49′26.54″N/ 002°46′46.78″W	R (2)
2	271	55°46′34.73″N/ 002°47′05.62″W	U (2)
3	360	55°47′24.57″N/ 002°43′31.04″W	R (2)
4	300	55°42′00.02″N/ 002°45′29.21″W	U (2)
5	232	55°44′28.64″N/ 002°38′33.57″W	R (2)
6	180	55°39′10.43″N/ 002°41′50.99″W	R + U (4)
7	100	55°41′36.40″N/ 002°42′32.59″W	U (2)
8	200	55°43′09.71″N/ 002°41′37.32″W	R (2)
9	230	55°44′09.73″N/ 002°40′25.34″W	R (2)
10	285	55°44′42.61″N/ 002°38′33.91″W	R + U (4)
11	195	55°43′59.74″N/ 002°46′10.80″W	U (2)
12	230	55°45′01.53″N/ 002°33′59.10″W	U (2)
13	207	55°45′32.21″N/ 002°28′43.98″W	R (2)
14	300	55°48′48.07″N/ 002°35′30.28″W	R (2)
15	208	55°48′23.28″N/ 002°30′06.09″W	R + U (4)
16	225	55°40′44.39″N/ 002°37′27.14″W	R + U (4)
17	140	55°39′59.73″N/ 002°33′15.31″W	R (2)
18	175	55°41′09.26″N/ 002°33′42.29″W	U (2)
19	180	55°42′51.82″N/ 002°32′54.37″W	R (2)
20	190	55°42′05.55″N/ 002°35′57.70″W	U (2)
21	247	55°52′05.45″N/ 002°25′54.64″W	R (2)
22	213	55°49′57.38″N/ 002°26′06.32″W	R (2)
23	180	55°50′21.93″N/ 002°22′53.91″W	U (2)
24	236	55°51′59.51″N/ 002°25′42.03″W	R + U (4)
25	120	55°55′13.74″N/ 002°20′45.68″W	U (2)
26*	180	55°45′10.70″N/ 002°24′30.61″W	R + U (4)
27*	100	55°45′38.64″N/ 002°21′06.79″W	R + U (4)
28	150	55°47′19.70″N/ 002°21′12.24″W	R + U (4)
29*	90	55°48′12.97″N/ 002°20′30.05″W	U (2)
30	135	55°41′34.10″N/ 002°25′04.03″W	R (2)

31	140	55°42′46.67″N/ 002°23′30.15″W	U (2)
32*	100	55°42′41.00″N/ 002°21′59.39″W	R + U (4)
33	60	55°40′58.23″N/ 002°21′03.75″W	U (2)
34	130	55°42′46.72″N/ 002°25′01.36″W	R (2)
35	220	55°55′03.85″N/ 002°15′55.46″W	R (2)
36	140	55°53′14.06″N/ 002°17′24.90″W	R + U (4)
37	130	55°52′48.31″N/ 002°12′25.08″W	U (2)
38	150	55°55′13.92″N/ 002°11′29.87″W	R (2)
39	150	55°54′23.37″N/ 002°10′22.08″W	U (2)

Table S2. Sampling locations for pure and mixed populations of *Geum rivale* (R) and *G. rivale* (U) within an area of geographic sympatry in Scotland. Numbers in brackets = total sample size. * indicates a population in which an early generation hybrid was detected.

Table S3. EcoRI P1 adapters and generic SphI P2 adapter; P1 and P2 primers used in ddRAD amplification

Oligo ID	EcoRI Adapter P1.1_Oligo sequence<br>EcoRI Adapter P1.2_Oligo sequence
ACACGACA_EcoRI_P1.Top	5'-ACACTCTTTCCCTACACGACGCTCTTCCGATCTACACGACA-3'
ACACGACA_EcoRI_P1.Bottom	5'-Phos-AATTTGTCGTGTAGATCGGAAGAGCGTCGTGTAGGGAAAGAGTGT-3'
ACGTAGCA_EcoRI_P1.Top	5'-ACACTCTTTCCCTACACGACGCTCTTCCGATCTACGTAGCA-3'
ACGTAGCA_EcoRI_Bottom	5'-Phos-AATTTGCTACGTAGATCGGAAGAGCGTCGTGTAGGGAAAGAGTGT-3'
ACTGCTCA_EcoRI_P1. Top	5'-ACACTCTTTCCCTACACGACGCTCTTCCGATCTACTGCTCA-3'
ACTGCTCA_EcoRI_P1.Bottom	5'-Phos-AATTTGAGCAGTAGATCGGAAGAGCGTCGTGTAGGGAAAGAGTGT-3'
AGCTGTGA_EcoRI_P1. Top	5'-ACACTCTTTCCCTACACGACGCTCTTCCGATCTAGCTGTGA-3'
AGCTGTGA_EcoRI_P1.Bottom	5'-Phos-AATTTCACAGCTAGATCGGAAGAGCGTCGTGTAGGGAAAGAGTGT-3'
AGTCACGA_EcoRI_P1. Top	5'-ACACTCTTTCCCTACACGACGCTCTTCCGATCTAGTCACGA-3'
AGTCACGA_EcoRI_P1.Bottom	5'-Phos-AATTTCGTGACTAGATCGGAAGAGCGTCGTGTAGGGAAAGAGTGT-3'
ATATCATA_EcoRI_P1. Top	5'-ACACTCTTTCCCTACACGACGCTCTTCCGATCTATATCATA-3'
ATATCATA_EcoRI_P1.Bottom	5'-Phos-AATTTATGATATAGATCGGAAGAGCGTCGTGTAGGGAAAGAGTGT-3'
CACACAGT_EcoRI_P1. Top	5'-ACACTCTTTCCCTACACGACGCTCTTCCGATCTCACACAGT-3'
CACACAGT_EcoRI_P1.Bottom	5'-Phos-AATTACTGTGTGAGATCGGAAGAGCGTCGTGTAGGGAAAGAGTGT-3'
CATGATCA_EcoRI_P1. Top	5'-ACACTCTTTCCCTACACGACGCTCTTCCGATCTCGATACTA-3'
CATGATCA_EcoRI_P1.Bottom	5'-Phos-AATTTGATCATGAGATCGGAAGAGCGTCGTGTAGGGAAAGAGTGT-3'
CGCGCATA_EcoRI_P1. Top	5'-ACACTCTTTCCCTACACGACGCTCTTCCGATCTCGCGCATA-3'

CGCGCATA_EcoRI_P1.Bottom	5'-Phos-AATTTATGCGCGAGATCGGAAGAGCGTCGTGTAGGGAAAGAGTGT-3’
CTAGTGTC_EcoRI_P1.Top	5' -ACACTCTTTCCCTACACGACGCTCTTCCGATCTCTAGTGTC-3’
CTAGTGTC_EcoRI_P1.Bottom	5'-Phos-AATTGACACTAGAGATCGGAAGAGCGTCGTGTAGGGAAAGAGTGT-3’
CTCTGCTC_EcoRI_P1. Top	5' -ACACTCTTTCCCTACACGACGCTCTTCCGATCTCTCTGCTC-3’
CTCTGCTC_EcoRI_P1.Bottom	5'-Phos-AATTGAGCAGAGAGATCGGAAGAGCGTCGTGTAGGGAAAGAGTGT-3’
CTGATGCT_EcoRI_P1. Top	5' -ACACTCTTTCCCTACACGACGCTCTTCCGATCTCTGATGCT-3’
CTGATGCT_EcoRIP1.Bottom	5'-Phos-AATTAGCATCAGAGATCGGAAGAGCGTCGTGTAGGGAAAGAGTGT-3’
GACTGCAG_EcoRI_P1. Top	5' -ACACTCTTTCCCTACACGACGCTCTTCCGATCTGACTGCAG-3’
GACTGCAG_EcoRI_P1.Bottom	5'-Phos-AATTCTGCAGTCAGATCGGAAGAGCGTCGTGTAGGGAAAGAGTGT-3’
GATCGTGA_EcoRI_P1. Top	5' -ACACTCTTTCCCTACACGACGCTCTTCCGATCTGATCGTGA-3’
GATCGTGA_EcoRI_P1.Bottom	5'-Phos-AATTTCACGATCAGATCGGAAGAGCGTCGTGTAGGGAAAGAGTGT-3’
GCATGTGC_EcoRI_P1. Top	5' -ACACTCTTTCCCTACACGACGCTCTTCCGATCTGCATGTGC-3’
GCATGTGC_EcoRI_P1.Bottom	5'-Phos-AATTGCACATGCAGATCGGAAGAGCGTCGTGTAGGGAAAGAGTGT-3’
GCTACAGC_EcoRI_P1 Top	5' -ACACTCTTTCCCTACACGACGCTCTTCCGATCTGCTACAGC-3’
GCTACAGC__EcoRI_P1.Bottom	5'-Phos-AATTGCTGTAGCAGATCGGAAGAGCGTCGTGTAGGGAAAGAGTGT-3’
GTACATCA_EcoRI_P1. Top	ACACTCTTTCCCTACACGACGCTCTTCCGATCTGTACATCA-3’
GTACATCA_EcoRI_P1.Bottom	5'-Phos-AATTTGATGTACAGATCGGAAGAGCGTCGTGTAGGGAAAGAGTGT-3’
GTGTACTG_EcoRI_P1 Top	5' -ACACTCTTTCCCTACACGACGCTCTTCCGATCTGTGTACTG-3’
GTGTACTG_EcoRI_P1.Bottom	5'-Phos-AATTCAGTACACAGATCGGAAGAGCGTCGTGTAGGGAAAGAGTGT-3’

TACGATAT_EcoRI_P1.Top	5' -ACACTCTTTCCCTACACGACGCTCTTCCGATCTTACGATAT
TACGATAT_EcoRI_P1.Bottom	5'-Phos-AATTATATCGTAAGATCGGAAGAGCGTCGTGTAGGGAAAGAGTGT-3’
TCAGCATC_EcoRI_P1. Top	5' -ACACTCTTTCCCTACACGACGCTCTTCCGATCTTCAGCATC-3’
TCAGCATC_EcoRI_P1.Bottom	5'-Phos-AATTGATGCTGAAGATCGGAAGAGCGTCGTGTAGGGAAAGAGTGT-3’
TCGAGTGA_EcoRI_P1 Top	5' -ACACTCTTTCCCTACACGACGCTCTTCCGATCTTCGAGTGA-3’
TCGAGTGA_EcoRI_P1.Bottom	5'-Phos-AATTTCACTCGAAGATCGGAAGAGCGTCGTGTAGGGAAAGAGTGT-3’
TCTCTCGA_EcoRI_P1. Top	5' -ACACTCTTTCCCTACACGACGCTCTTCCGATCTTCTCTCGA-3’
TCTCTCGA_EcoRI_P1.Bottom	5'-Phos-AATTTCGAGAGAAGATCGGAAGAGCGTCGTGTAGGGAAAGAGTGT-3’
TGCACTAC_EcoRI_P1. Top	5' -ACACTCTTTCCCTACACGACGCTCTTCCGATCTTGCACTAC-3’
TGCACTAC_EcoRI_P1.Bottom	5'-Phos-AATTGTAGTGCAAGATCGGAAGAGCGTCGTGTAGGGAAAGAGTGT-3’
TGTGACTG_EcoRI_P1. Top	5' -ACACTCTTTCCCTACACGACGCTCTTCCGATCTTGTGACTG-3’
TGTGACTG_EcoRI_P1.Bottom	5'-Phos-AATTCAGTCACAAGATCGGAAGAGCGTCGTGTAGGGAAAGAGTGT-3’
**Oligo ID**	**SphI Adapter P2.1_Oligo sequence**<br>**SphI Adapter P2.2_Oligo sequence**
SphI-P2_Top	5’- Phos-AGATCGGAAGAGCGAGAACAA-3’
SphI-P2_Bottom	5'-GTGACTGGAGTTCAGACGTGTGCT CTTCCGATCTCATG-3'

Oligo ID	P1 PCR primer_Oligo sequence
P1 PCR primer	5'-AATGATACGGCGACCACCGAGATCTACACTCTTTCCCTACACGACG-3'
**Oligo ID**	**P2 PCR primer_Oligo sequence**
ddRAD_PCR2_Idx_AACGTGAT	5'- CAAGCAGAAGACGGCATACGAGATAACGTGATGTGACTGGAGTTCAGACGTGTGC-3'
ddRAD_PCR2_Idx_CGCTGATC	5'- CAAGCAGAAGACGGCATACGAGATCGCTGATCGTGACTGGAGTTCAGACGTGTGC-3'
ddRAD_PCR2_Idx_ACAAGCTA	5'- CAAGCAGAAGACGGCATACGAGATACAAGCTAGTGACTGGAGTTCAGACGTGTGC-3'
ddRAD_PCR2_Idx_CTGTAGCC	5'- CAAGCAGAAGACGGCATACGAGATCTGTAGCCGTGACTGGAGTTCAGACGTGTGC-3'
ddRAD_PCR2_Idx_ACGCTCGA	5'- CAAGCAGAAGACGGCATACGAGATACGCTCGAGTGACTGGAGTTCAGACGTGTGC-3'
ddRAD_PCR2_Idx_ ACGTATCA	5'- CAAGCAGAAGACGGCATACGAGATACGTATCAGTGACTGGAGTTCAGACGTGTGC -3'
ddRAD_PCR2_Idx_ ACTATGCA	5'- CAAGCAGAAGACGGCATACGAGATACTATGCAGTGACTGGAGTTCAGACGTGTGC -3'
ddRAD_PCR2_Idx_AGAGTCAA	5'- CAAGCAGAAGACGGCATACGAGATAGAGTCAAGTGACTGGAGTTCAGACGTGTGC -3'
ddRAD_PCR2_Idx_AGATCGCA	5'- CAAGCAGAAGACGGCATACGAGATAGATCGCAGTGACTGGAGTTCAGACGTGTGC-3'
ddRAD_PCR2_Idx_AGCAGGAA	5'- CAAGCAGAAGACGGCATACGAGATAGCAGGAAGTGACTGGAGTTCAGACGTGTGC-3'
ddRAD_PCR2_Idx_AGTCACTA	5'- CAAGCAGAAGACGGCATACGAGATAGTCACTAGTGACTGGAGTTCAGACGTGTGC-3'
ddRAD_PCR2_Idx_ATCCTGTA	5'- CAAGCAGAAGACGGCATACGAGATATCCTGTAGTGACTGGAGTTCAGACGTGTGC-3'
ddRAD_PCR2_Idx_ATTGAGGA	5'- CAAGCAGAAGACGGCATACGAGATATTGAGGAGTGACTGGAGTTCAGACGTGTGC-3'
ddRAD_PCR2_Idx_CACTTCGA	5'- CAAGCAGAAGACGGCATACGAGATCACTTCGAGTGACTGGAGTTCAGACGTGTGC -3'
ddRAD_PCR2_Idx_CAGCGTTA	5'- CAAGCAGAAGACGGCATACGAGATCAGCGTTAGTGACTGGAGTTCAGACGTGTGC -3'
ddRAD_PCR2_Idx_CATACCAA	5'- CAAGCAGAAGACGGCATACGAGATCATACCAAGTGACTGGAGTTCAGACGTGTGC -3'
ddRAD_PCR2_Idx_CCAGTTCA	5'- CAAGCAGAAGACGGCATACGAGATCCAGTTCAGTGACTGGAGTTCAGACGTGTGC -3'
ddRAD_PCR2_Idx_GTGTTCTA	5'- CAAGCAGAAGACGGCATACGAGATGTGTTCTAGTGACTGGAGTTCAGACGTGTGC-3'
ddRAD_PCR2_Idx_GCTCGGTA	5'- CAAGCAGAAGACGGCATACGAGATGCTCGGTAGTGACTGGAGTTCAGACGTGTGC -3'
ddRAD_PCR2_Idx_GGAGAACA	5'- CAAGCAGAAGACGGCATACGAGATGGAGAACAGTGACTGGAGTTCAGACGTGTGC -3'
ddRAD_PCR2_Idx_GTGTTCTA	5'- CAAGCAGAAGACGGCATACGAGATGTGTTCTAGTGACTGGAGTTCAGACGTGTGC -3'
ddRAD_PCR2_Idx_TAGGATGA	5'- CAAGCAGAAGACGGCATACGAGATTAGGATGAGTGACTGGAGTTCAGACGTGTGC -3'
ddRAD_PCR2_Idx_TGGCTTCA	5'- CAAGCAGAAGACGGCATACGAGATTGGCTTCAGTGACTGGAGTTCAGACGTGTGC-3'
ddRAD_PCR2_Idx_TTCACGCA	5'- CAAGCAGAAGACGGCATACGAGATTTCACGCAGTGACTGGAGTTCAGACGTGTGC-3'
ddRAD_PCR2_Idx_GCCAAGAC	5'- CAAGCAGAAGACGGCATACGAGATGCCAAGACGTGACTGGAGTTCAGACGTGTGC-3'

	Unfiltered for paralogs	
Population by species	Number of SNPs	F_{IS}
G. rivale		
Ben Lawers	2531	-0.0082
Coire Garblach	2605	0.0384
Ben Lui	2664	0.0423
G. urbanum		
Priory Wood	551	0.6216
Mill Wood	531	-0.0697
Burgh Wood	542	0.5913
Hoades Wood	451	0.5100
Punnetts Town	495	0.4636
Frith Wood	507	0.6134
Stanford Bridge	535	0.6112
Combe Wood	536	0.6110
Copperhurst	509	0.5874
Selwyn Wood	166	-0.4759

Table S4. F_{IS} estimates for 'allopatric' UK *G. rivale* and *G. urbanum* populations using data not filtered for paralogs.

Sample	Heterozygosity	No. SNPs private to *G. rivale*	No. SNPs private to *G. urbanum*	No. SNPs shared by both species	No. alternately fixed SNPs	No. DNA blocks
Ben Lui 1		250	88	0	1184	668
Ben Lui 4		299	86	1	1170	672
Ben Lawers 5		179	88	0	1189	649

Table S5: Polymorphism counts and numbers of DNA blocks for coalescent analysis for three individual *G. rivale* samples from UK allopatric populations.

	Model		
	div_2	$IM_{r \rightarrow u}$	$IM_{u \rightarrow r}$
Ben Lui 1			
$N_{anc} = N_{rivb}$	$1.03 * 10^5$	$1.01 * 10^5$	$6.42 * 10^4$
N_{urb}	$3.46 * 10^4$	$3.23 * 10^4$	$3.29 * 10^4$
t (years)	$2.36 * 10^6$	$2.41 * 10^6$	$3.03 * 10^6$
M		0.02 (0.00 – 0.18)	0.08 (0.06 – 0.11)
Ben Lui 4			
$N_{anc} = N_{rivb}$	$1.30 * 10^5$	$1.24 * 10^5$	$1.11 * 10^5$
N_{urb}	$3.77 * 10^4$	$3.09 * 10^4$	$3.38 * 10^4$
t (years)	$2.22 * 10^6$ ($2.01 * 10^6$ – $2.43 * 10^6$)	$2.35 * 10^6$	$2.48 * 10^6$
M (mean, 95% CI's)		0.04 (0.005 - 0.191)	0.04 (0.007 - 0.101)
Ben Lawers 5			
$N_{anc} = N_{rivb}$	$7.41 * 10^4$	$7.34 * 10^4$	$5.11 * 10^4$
N_{urb}	$3.54 * 10^4$	$3.40 * 10^4$	$3.38 * 10^4$
t (years)	$2.60 * 10^6$	$2.64 * 10^6$	$3.10 * 10^6$
M (mean, 95% CI's)		0.01 (0.00 – 0.103)	0.02 (0.002 - 0.052)

Table S6 Parameter estimates under models analysed for sample pairs involving the samples: Ben Lui 1, Ben Lui 4, and Ben Lawers 5. 95% Confidence intervals provided in parentheses. See Figure S4 for plots of 95% CI's.

SUPPLEMENTARY FIGURES

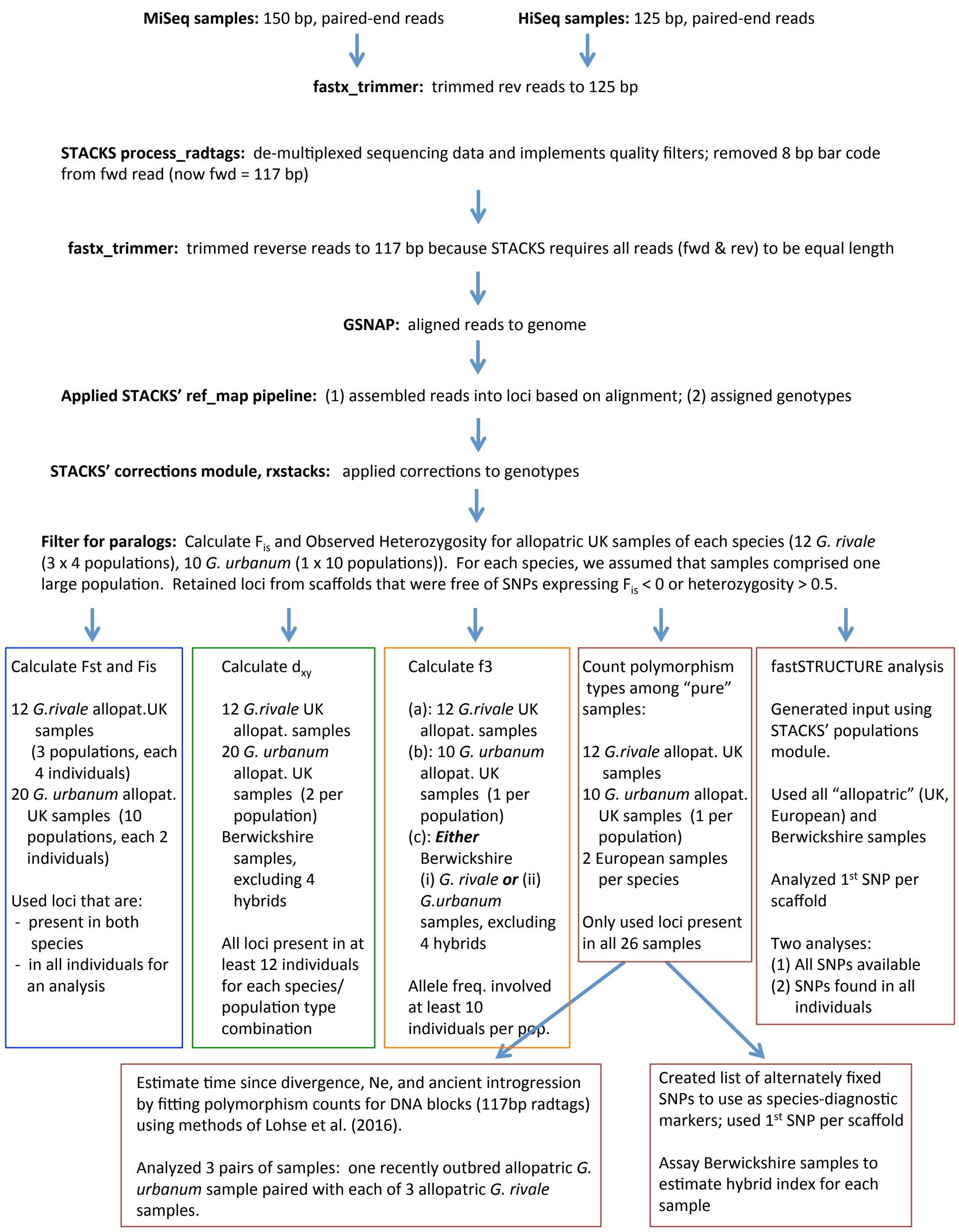

Figure S1: Bioinformatics pipeline for analysis of *Geum* ddRAD sequences.

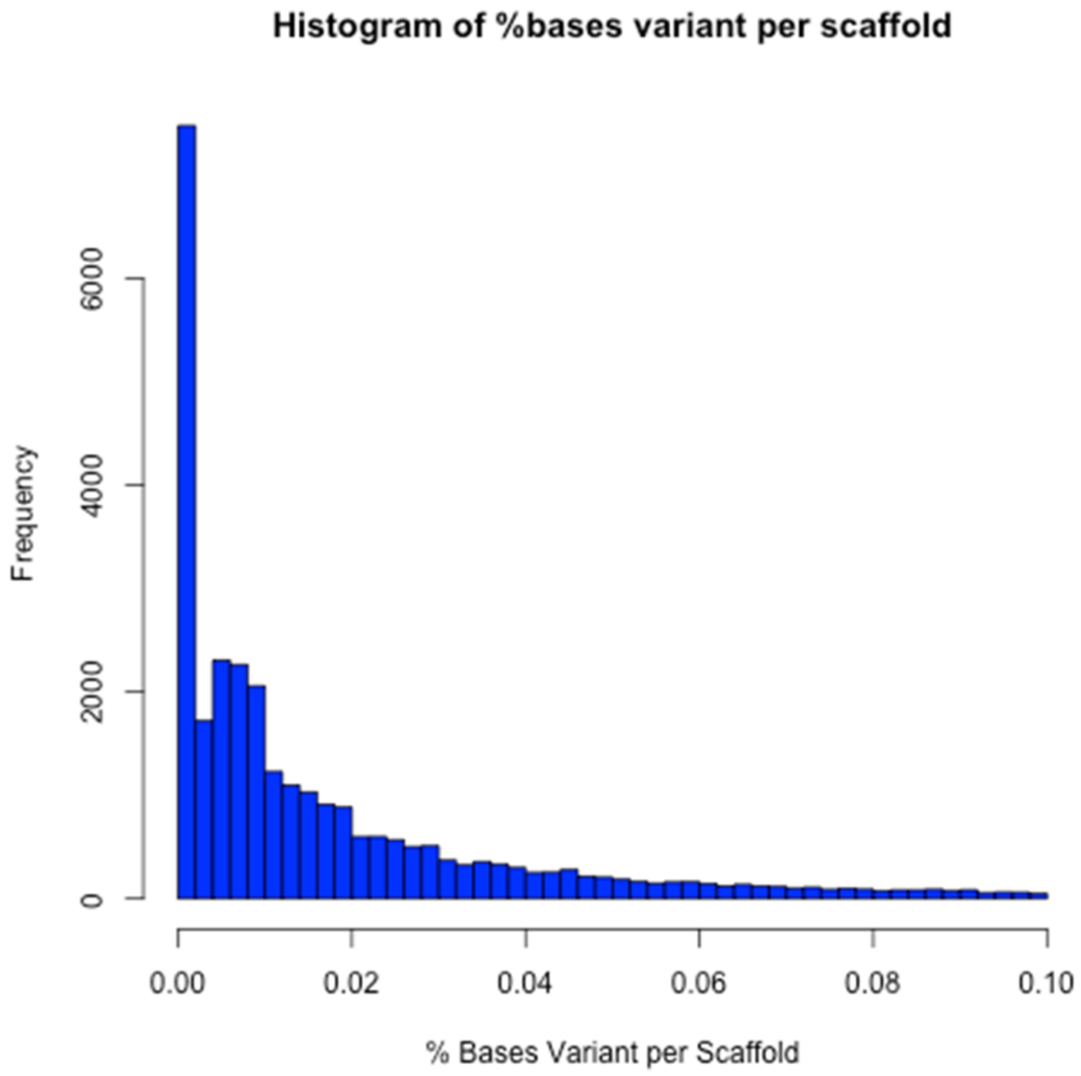
Histogram of %bases variant per scaffold
Frequency
0
2000
4000
6000
0.00
0.02
0.04
0.06
0.08
0.10
% Bases Variant per Scaffold

Figure S2.

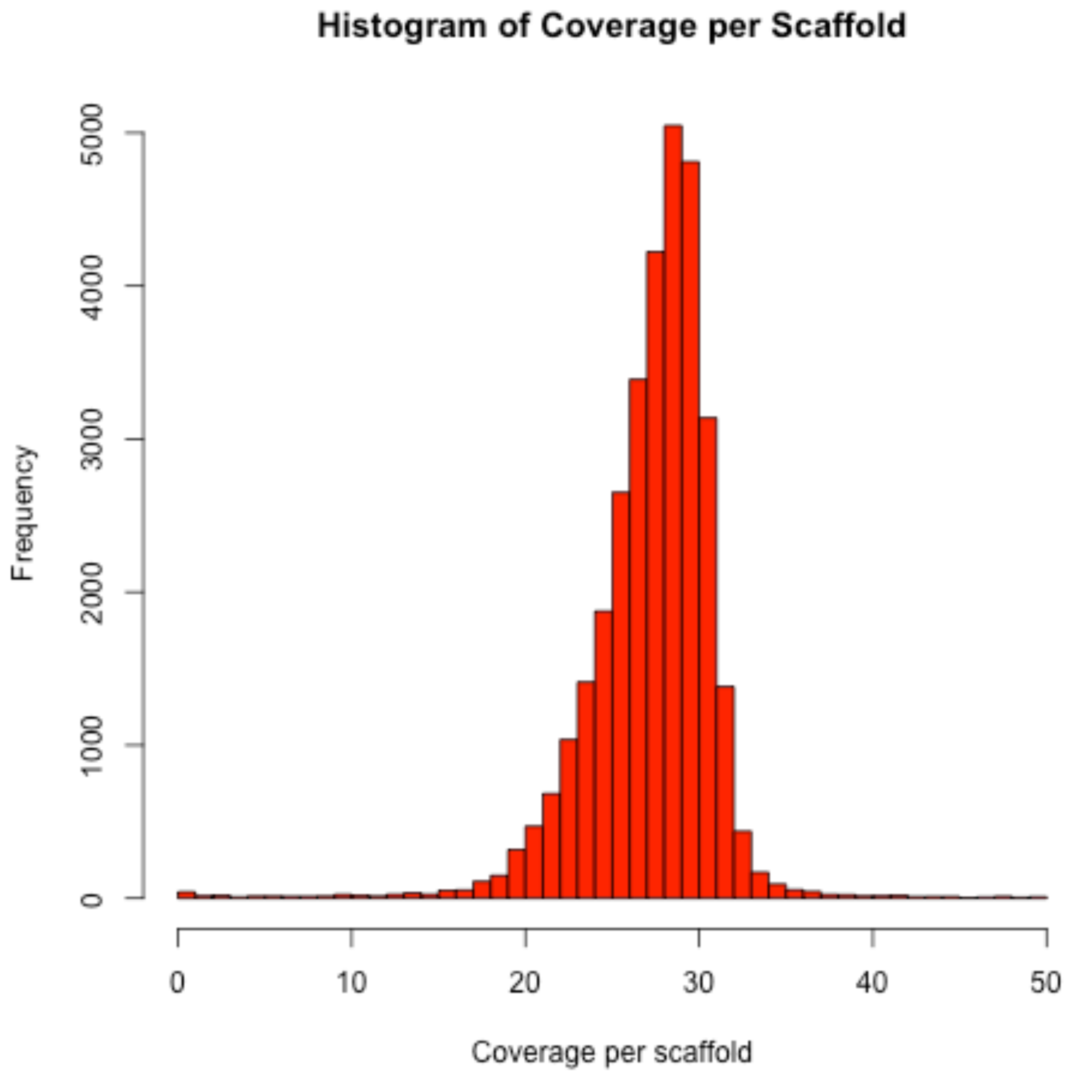
Histogram of Coverage per Scaffold
Frequency
0
1000
2000
3000
4000
5000
0
10
20
30
40
50
Coverage per scaffold

Figure S3

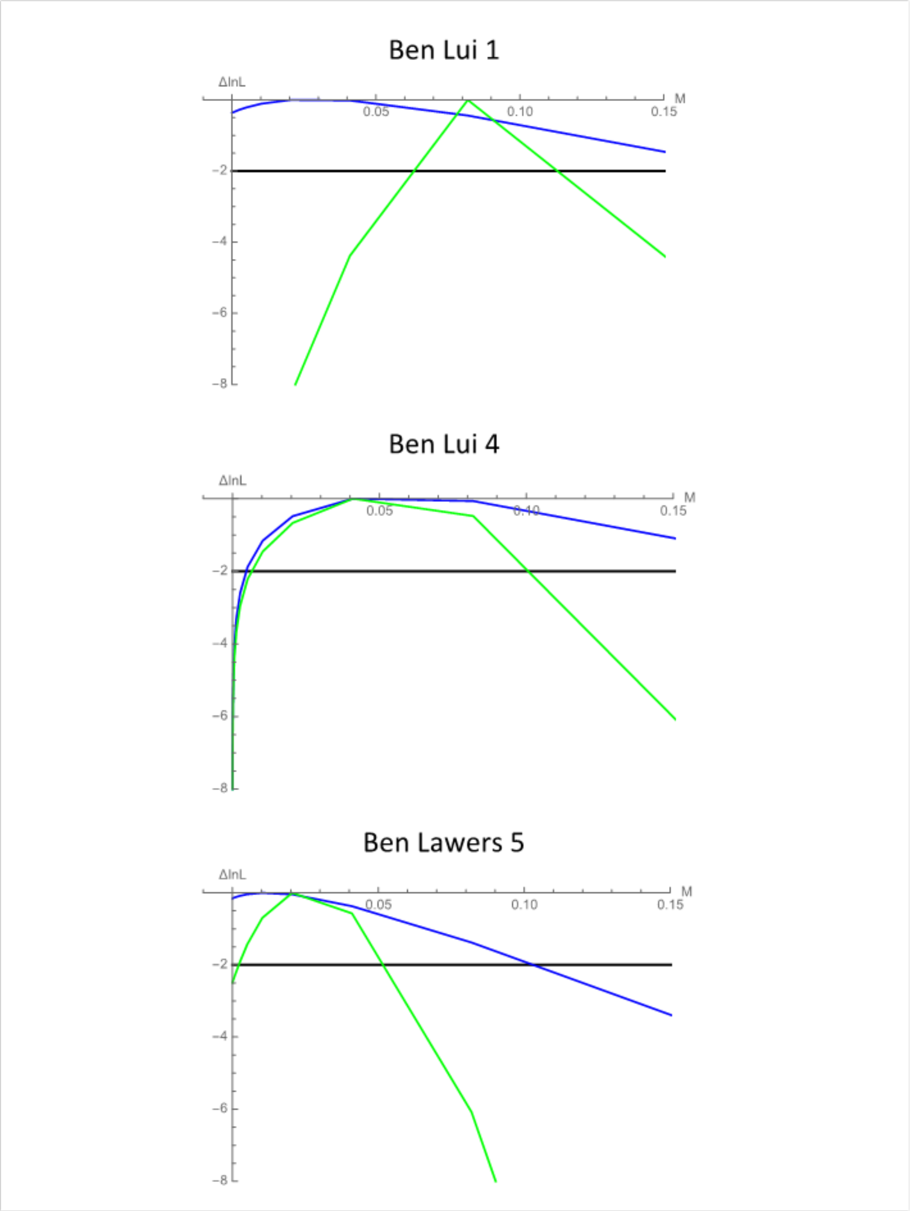

Figure S4: Log-Likelihood plots for estimates of M for three pairs of *Geum* samples, each involving a different *G. rivale* individual (Ben Lui 1, Ben Lui 4, Ben Lawers 5). Blue and green lines represent log-likelihoods of M for models $IM_{r \rightarrow u}$ and $IM_{u \rightarrow r}$, respectively. 95% CI's are bounded where these lines intersect the dark horizontal line.

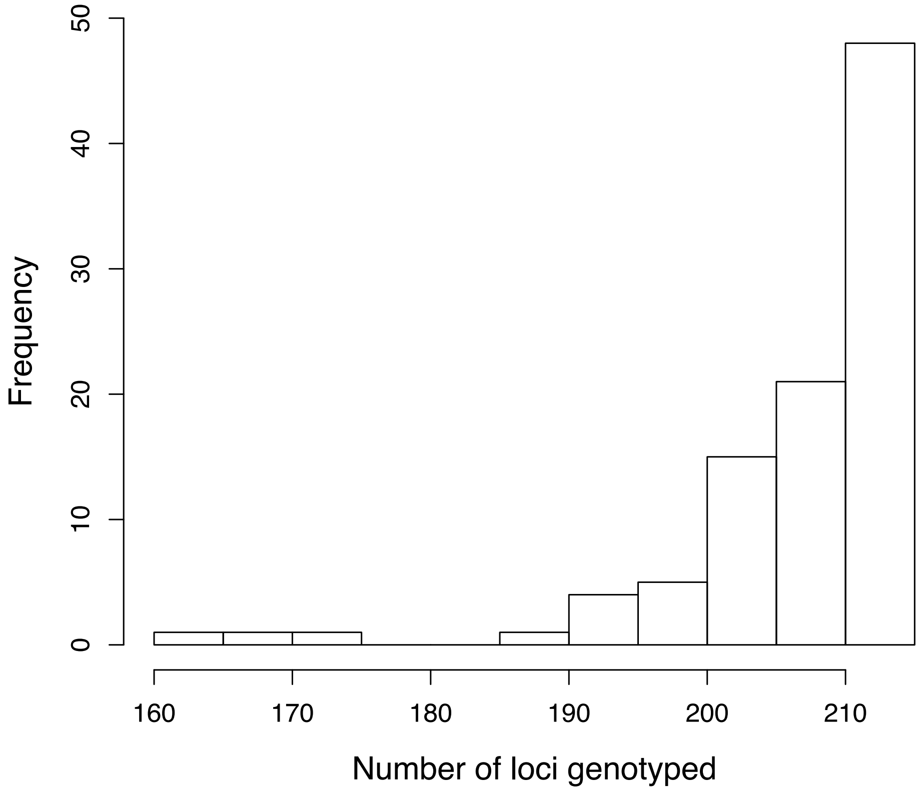

Figure S5. Number of species-specific SNPs scored per individual in a sample of 96 *Geum* plants from 39 populations in Berwickshire.

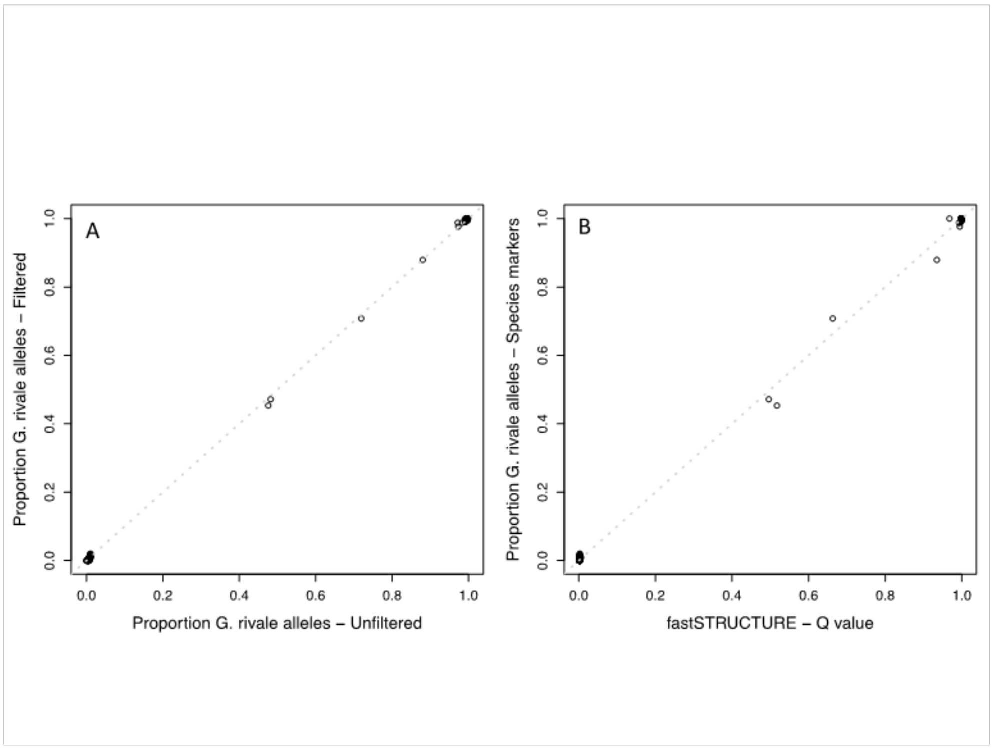

Figure S6. Genomic composition estimates for Berwickshire samples derived from paralog-filtered species-diagnostic SNPs (y-axis in both panels) compared to analogous measures in two alternate analyses: (A) species-diagnostic SNPs from data unfiltered for paralogs, and (B) Admixture proportion (Q value) calculated by fastSTRUCTURE analysis that required loci to be present in all individuals (188 SNPs).

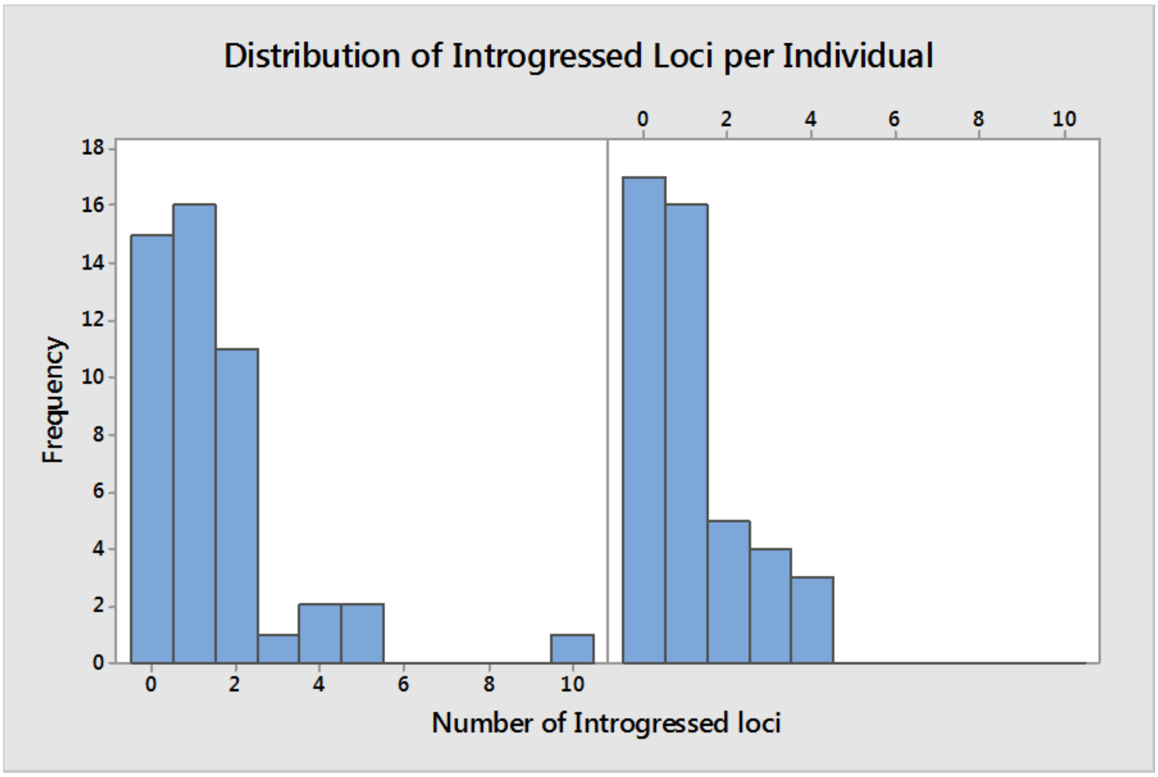

Figure S7. Number of putatively introgressed loci per individual in individuals classified as *G. rivale* (left panel) and *G. urbanum* (right panel) sampled from 39 populations in Berwickshire.
